# Prediction of severe adverse events, modes of action and drug treatments for COVID-19’s complications

**DOI:** 10.1038/s41598-021-00368-6

**Published:** 2021-10-21

**Authors:** Courtney Astore, Hongyi Zhou, Joshy Jacob, Jeffrey Skolnick

**Affiliations:** 1 Center for the Study of Systems Biology, School of Biological Sciences, Georgia Institute of Technology, 950 Atlantic Drive, N.W., Atlanta, GA 30332 USA; 2 Emory Vaccine Center, Emory University, Atlanta, GA 30329 USA; 3 Yerkes National Primate Research Center, Emory University, Atlanta, GA 30329 USA; 4 Department of Microbiology and Immunology, Emory Vaccine Center, School of Medicine, Emory University, Atlanta, GA 30329 USA

**Keywords:** Computational models, Machine learning, Virtual drug screening, Drug screening, Target identification, Computational biology and bioinformatics, Drug discovery, Molecular medicine, Signs and symptoms

## Abstract

Following SARS-CoV-2 infection, some COVID-19 patients experience severe host driven adverse events. To treat these complications, their underlying etiology and drug treatments must be identified. Thus, a novel AI methodology MOATAI-VIR, which predicts disease-protein-pathway relationships and repurposed FDA-approved drugs to treat COVID-19’s clinical manifestations was developed. SARS-CoV-2 interacting human proteins and GWAS identified respiratory failure genes provide the input from which the mode-of-action (MOA) proteins/pathways of the resulting disease comorbidities are predicted. These comorbidities are then mapped to their clinical manifestations. To assess each manifestation’s molecular basis, their prioritized shared proteins were subject to global pathway analysis. Next, the molecular features associated with hallmark COVID-19 phenotypes, e.g. unusual neurological symptoms, cytokine storms, and blood clots were explored. In practice, 24/26 of the major clinical manifestations are successfully predicted. Three major uncharacterized manifestation categories including neoplasms are also found. The prevalence of neoplasms suggests that SARS-CoV-2 might be an oncovirus due to shared molecular mechanisms between oncogenesis and viral replication. Then, repurposed FDA-approved drugs that might treat COVID-19’s clinical manifestations are predicted by virtual ligand screening of the most frequent comorbid protein targets. These drugs might help treat both COVID-19’s severe adverse events and lesser ones such as loss of taste/smell.

## Introduction

The COVID-19 pandemic is caused by SARS-CoV-2, a positive-sense, single-stranded, rapidly mutating RNA coronavirus^1^. The societal impact of COVID-19 is amplified by the minority of individuals experiencing significant complications/death. These include acute respiratory distress syndrome^2^, clotting issues, cytokine storms, hypoxemia, low white blood cell counts, bone marrow failure^3–6^ as well as less severe complications including loss of smell/taste and/or unusual neurological symptoms^7, 8^. Despite the development of COVID-19 vaccines, until herd immunity is reached, there will be new cases of COVID-19 with its resulting complications, in addition to the long-term effects of COVID-19^9^.

The primary objective of this work is to identify the molecular mechanisms and possible repurposed FDA-approved drug treatments for COVID-19’s clinical manifestations. This is a first step that suggests which drugs should be subsequently tested in a clinical setting. Repurposed drugs might treat the dual aspects of COVID-19 infections: The first approach directly attacks SARS-CoV-2 to kill the virus, e.g. Remdesivir^6^. The second approach, and the goal of this contribution, is to develop treatments for the downstream, post-infection clinical manifestations. Based on media coverage, one might be under the mistaken impression that all drugs have been tested for COVID-19 repurposing^10^. In reality, this is not true. As shown in the Supplementary Information (SI) Tables S1 and S2, for the 24 mapped complications addressed here, the average number of FDA-approved drugs undergoing clinical trials within the top 20 predicted drugs is < 5. Five complications lack any drugs in clinical trials. Thus, which repurposed drugs might treat a given patient’s severe adverse reactions is yet unknown. Clearly, a systematic method to identify effective repurposed drugs is preferred over a random, anecdotal approach.

With that goal, we developed a new algorithm, MOATAI-VIR, Mode-Of-Action proteins and Targeted therapeutic discovery driven by Artificial Intelligence for VIRuses designed to predict the mode-of-action (MOA) proteins of COVID-19’s severe patient responses based on predicted COVID-19 disease comorbidities. It then suggests repurposed drugs to help prevent or mitigate COVID-19’s severe complications. To accomplish this, we input either the experimentally determined human-SARS-CoV-2 interactome or COVID-19 GWAS survival-associated risk genes as a MOA indication profile^5^. These profiles are used to determine the disease comorbidities associated with the MOA proteins presumably causing a particular complication. In practice, each COVID-19 comorbid disease is mapped to its respective clinical manifestation group in^11^, which provides 30 respiratory and non-respiratory COVID-19 in-hospital clinical complications. Then, the top comorbidity enriched MOA proteins are subject to pathway analysis to identify the underlying molecular processes. There were also comorbidities that did not map to a characterized COVID-19 clinical manifestation; one example is cancers. The set of comorbid diseases or their most frequent protein targets are used to suggest possible repurposed drug treatments.

## Results

### Prediction of COVID-19’s complications and underlying molecular mechanisms

**Figure 1:**
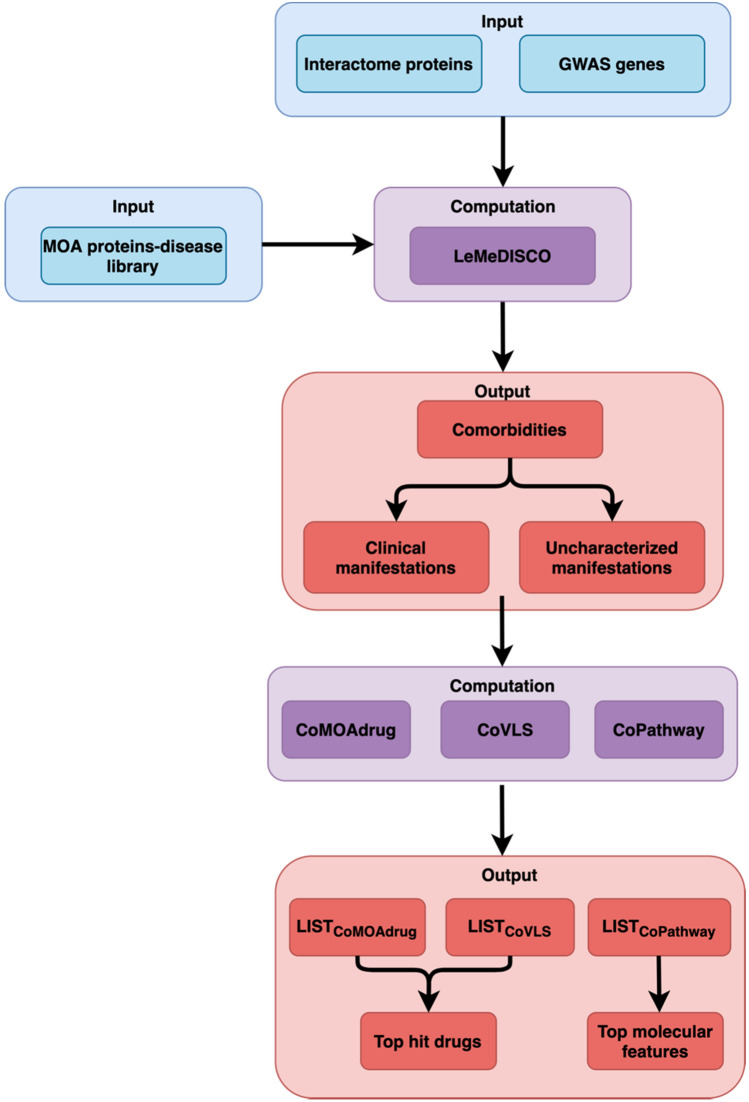
Overview of the MOATAI-VIR approach that predicts comorbid human diseases, their MOA proteins, and repurposed drugs to address the severe secondary adverse events. Blue are MOATAI-VIR inputs, purple algorithms and pink output predictions.

An overview of MOATAI-VIR is shown in Fig. 1, with a more detailed flowchart in Fig. S1. The goal of MOATAI-VIR is to identify the human mode-of-action proteins responsible for the severe adverse responses associated with COVID-19. This information is then used to predict FDA-approved drugs that treat these complications. To accomplish this, we input either the experimentally determined human proteins from the human-SARS-CoV-2 interactome^6^ or COVID-19 GWAS survival associated risk genes^2, 5^ as MOA profiled in MEDICASCY. We then employ our recently developed LeMeDISCO algorithm which predicts disease comorbidity and the molecular interactions responsible for the severe adverse events resulting from SARS-CoV-2 infection.

### Large scale benchmarking of MEDICASCY MOA predictions

The first step uses MEDICASCY^12^ to predict MOA protein targets of all diseases. For large scale benchmarking for MOA prediction, we map all drugs in our indication library to DrugBank drugs^13^ (v5.09) and obtain their respective human protein targets. These are combined with those from the Therapeutic Target Database^14^. Using drug-indication relationships in our training library, we compiled indication-protein target relationships of 145,722 pairs for 3539 indications (with an average ~ 41 proteins/indication) for benchmarking. In benchmarking, any drug in the training library having a Tanimoto coefficient, Tc^15^ ≥ 0.8 to the given drug whose indications are predicted is excluded. We define a MOA prediction for an indication when its *p*-value < 0.05 using the upper tailed null hypothesis. 43.7% of indications have correctly predicted protein-indications. If a Tc = 1 cutoff in training is used, this increases to 65.9%. However, the incompleteness of the known MOA targets suggests that these are lower bounds.

### Benchmarking of LeMeDISCO

As discussed in SI, Table S0, large scale benchmarking of LeMeDISCO on different clinical data sets shows high comorbid disease coverage and accuracy compared to alternative methods^16–19^. It is also superior to alternatives that rely solely on symptom data, which lack molecular mechanism-based associations and cannot provide information of confidence-ranked putative protein targets. In practice, LeMeDISCO’s recall rate is close to 70% for a representative set of 2630 disease pairs.

### COVID-19’s clinical manifestations

We first predicted COVID-19 comorbidities using the 332 high confidence human proteins that interact with SARS-CoV-2^6^. There are 916 significant comorbidities (with a *p*-value cutoff < 0.05), of which 458 map to a COVID-19 clinical manifestation group. The top two disease comorbidities ranked by their p-value are shown in Table 1, with an expanded list in Table S1. Also provided are comorbidity enriched protein targets. Without extrinsic information or training, MOATAI-VIR recapitulates many key COVID-19 phenotypes such as myelosuppression, immunodeficiency, neurotoxicity, blood indications, myocardial infarctions, stroke, and cytokine storm symptoms^2, 6, 20^. The ICD-10 code of the comorbid diseases was used to map them to the 30 complications in^11^. In practice, indications are mapped to 21/30 COVID-19 complications (see Table 1). Our library of 3608 indications^10^ does not have these 4/30 complications: Dialysis initiation, Intracranial hemorrhage, Hypertensive crisis, Cardiogenic shock. Thus, the complication recall rate is 21/26 ~ 81%. These mapped indications are then used to prioritize MOA proteins, pathways, and predict drugs for each complication.

**Table 1:** Top 2 comorbidities, comorbidity enriched MOA proteins, top pathway, and top 2 repurposed FDA drugs predicted to treat specific COVID-19 severe adverse clinical manifestations using the SARS-CoV-2 interactome as input.

Clinical manifestation	Comorbidities	Comorbidity enriched MOA proteins	Top pathway	CoMOAdrug drugs	CoVLS drugs
Neurologic	Aseptic meningitis toxic encephalopathy	C5orf52 L3HYPDH	Glutathione conjugation	Pomalidomide Tetrahydrofolic acid	Gadobutrol Amphetamine*
Respiratory	Severe acute respiratory syndrome viral pneumonia	MYH8 MYH2	FCGR3A-mediated phagocytosis	Prednisolone*^a^ Tetrahydrofolic acid	Delafloxacin* Gatifloxacin
Hematologic	Uveal cancer Fanconi anemia	GMPR ASMTL	RAB geranylgeranylation	Vindesine Pomalidomide*	Emtricitabine^a^ Lamivudine*
Endocrine	Ovarian disease lysosomal storage disease	GMPR NTPCR	RAB geranylgeranylation	Vindesine Pomalidomide	Emtricitabine^a^ Lamivudine
Ocular symptoms	Neuroretinitis retinal artery	MYH8 MYO7A	Nuclear Receptor transcription pathway	Prednisolone*^a^ Tetrahydrofolic acid	Diflorasone Delafloxacin
Renal acute kidney failure injury	Perineurioma Fanconi syndrome	RETSAT RDH14	RAB geranylgeranylation	Pomalidomide* Vindesine	Citric* Acid Emtricitabine*^a^
Cardiovascular/arrhythmia	Coronary stenosis brain infarction block	GMPR ASMTL	RAB geranylgeranylation	Pomalidomide Vindesine	Emtricitabine*^a^ Lamivudine*
Sepsis	Hepatitis A/D/E hantavirus	ASMTL TUBE1	RAB geranylgeranylation	Vindesine Pomalidomide	Emtricitabine*^a^ Lamivudine*
Hepatocellular injury/acute hepatitis/liver failure	Biliary tract disease exocrine pancreatic insufficiency	ASMTL DGUOK	RAB geranylgeranylation	Cabazitaxel Lactulose	Emtricitabine^a^ Lamivudine
Cerebral ischemia/infarction	Brain infarction lymphatic system disease	RPS19 POLR3F	Activation of gene expression by SREBF (SREBP)	Pomalidomide Ifosfamide	Phenyl salicylate Emtricitabine^a^
Gastrointestinal symptoms	Exanthema hemoglobinuria chronic fatigue	ASMTL TUBE1	RAB geranylgeranylation	Tetrahydrofolic acid Cabazitaxel	Emtricitabine^a^ Vindesine
Bacteremia	Exanthema hemoglobinuria chronic fatigue	ASMTL TUBE1	RAB geranylgeranylation	Tetrahydrofolic acid Cabazitaxel	Emtricitabine^a^ Vindesine
Dermatologic complications/pressure ulcer	Diffuse scleroderma phototoxic dermatitis rosacea	COX7A2L COX7A1	Folding of actin by CCT/TriC	Pomalidomide Vindesine	Diflorasone Betamethasone
Respiratory failure	Respiratory system disease	MYH8 MYH2	Leishmania phagocytosis	Acarbose Amoxicillin^a^	Delafloxacin Gatifloxacin
Pulmonary embolism	Pulmonary embolism and infarction	C5orf52 L3HYPDH	Glutathione conjugation		Gadobutrol Amphetamine
Pneumothorax	Spontaneous tension pneumothorax	MYH8 MYH2	Leishmania phagocytosis	Acarbose Amoxicillin^a^	Delafloxacin Gatifloxacin
Pneumonia	Pleuropneumonia	SLC8A3 KCNA10	Nuclear Receptor transcription pathway	Prednisolone*^a^ Prednisone*$	Betamethasone* Norelgestromin
DIC	Purpura fulminans	RPS19 TCOF1	TWIK-releated acid-sensitive K + channel	Prednisolone^a^ Estriol	Isocarboxazid Triflusal Calcium
Asthma exacerbation	Cough variant asthma	MYH8 MYH2	Leishmania phagocytosis	Acarbose Amoxicillin^a^	Delafloxacin Gatifloxacin
Acute myocardial infarction	Coronary thrombosis	ATP5D GLRA1	MECP2 regulates neuronal receptors and channels	Acarbose Clarithromycin^a^	Halothane Methoxyflurane*
ARDS	Adult respiratory distress syndrome	RETSAT KCNA10	RAB geranylgeranylation	Tetrahydrofolic acid Vindesine	Betamethasone* Levonorgestrel

Current FDA-approved drugs are based on the top 20 drug predictions ranked by CoMOAdrug or CoVLS for complications. Drugs with side effects predicted by MEDICASCY which is the same as the complications were excluded.

*Indicates the drug is currently undergoing clinical trials for the COVID-19 complication in column 1.

^a^Are drugs under trial for a general COVID-19 patient. The top 20 list of comorbidity ranked drugs are in SI, Table S1.

The 6 human genes near the 3p21.31 locus of the human genome identified in a GWAS study as strongly associated with respiratory failure in COVID-19 patients^5^ (odds ratio 1.77) were next used to predict comorbidities. We determined 598 significant comorbidities having a *p*-value < 0.05, of which 360 map to a clinical manifestation group. As shown in Table 2, many severe clinical complications associated with COVID-19 are predicted including respiratory complications, myocardial infarction, and cytokine storms. Table S2 provides an expanded list including myocardial infarction, stroke, neurological manifestations, hearing disorders, hypoxemia, lung, cardiovascular and diabetic risk factors^2, 20, 21^. Excluding 4 indications not in our library, with GWAS risk gene input, the recall rate of the COVID-19 complications is also 21/26 ~ 81%. Since the 332 proteins of the SARS-CoV-2 human interactome and the 6 GWAS COVID-19 survival risk gene do not overlap except for FYCO1, their comorbidity predictions are partially complementary. Combining both the human interactome and GWAS complications predicts 24/26 (92%) COVID-19 complications.

**Table 2:** Top 2 comorbidities, comorbidity enriched MOA proteins, top pathway, and top 2 repurposed FDA drugs predicted to treat specific COVID-19 severe adverse clinical manifestations using GWAS input.

Clinical manifestation	Comorbidities	Comorbidity enriched MOA proteins	Top pathway	CoMOAdrug drugs	CoVLS drugs
Neurologic	Congenital myasthenic syndrome myotonia congenita	C5orf52 L3HYPDH	Glutathione conjugation	Codeine* Paliperidone*	Amphetamine* Mephentermine
Cardiovascular/Arrhythmia	Carotid stenosis brain ischemia	APLNR ADRB3	Class A/1 (Rhodopsin-like receptors)	Codeine* Trandolapril*	Modafinil Mephentermine
Endocrine	Nodular goiter hyperinsulinemic hypoglycemia	COX7A1 COX7A2L	Olfactory Signaling Pathway	Prasterone Prednisolone*^a^	Betamethasone* Levonorgestrel*
Ocular symptoms	Auditory system disease ocular hyperemia	NR4A3 NR3C2	Nuclear Receptor transcription pathway	Prednisolone*^a^ Framycetin	Diflorasone Betamethasone*
Respiratory	Upper respiratory tract disease lower respiratory tract disease	COX7A1 COX7A2L	Nuclear Receptor transcription pathway	Prednisolone*^a^ Codeine*	Betamethasone* Deflazacort
Hepatocellular injury/Acute hepatitis/liver failure	Biliary dyskinesia acalculous cholecystitis	CX3CR1 OR51V1	Olfactory signaling pathway	Codeine Prasterone Drostanolone	Ipratropium bromide Propantheline
Renal/Acute kidney failure injury	Gynecomastia premenstrual tension	SLC8A3 ELOVL7	Nuclear Receptor transcription pathway	Dicloxacillin Piperacillin*^a^	Levonorgestrel Citric* acid
Cerebral ischemia/infarction	Carotid stenosis brain ischemia	PTGDR2 APLNR	Class A/1 (Rhodopsin-like receptors)	Codeine Acarbose	Naltrexone^a^ Salbutamol
Acute myocardial infarction	Myocardial infarction	ADRB3 SLC6A16	Class A/1 (Rhodopsin-like receptors)	Trandolapril Prednisolone*^a^	Modafinil Mephentermine
Hematologic	Methemoglobinanemia hemorrhagic disease	COX7A2L COX7A1	Olfactory signaling pathway	Prasterone Prednisolone*^a^	Drostanolone Levonorgestrel*
Gastrointestinal symptoms	Sexual dysfunction alexia	SLC6A20 SLC6A16	Amine ligand-binding receptors	Tetrahydrofolic acid Cabazitaxel	Modafinil* Mephentermine
Bacteremia	Sexual dysfunction alexia	SLC6A20 SLC6A16	Amine ligand-binding receptors	Tetrahydrofolic acid Cabazitaxel	Modafinil Mephentermine
Sepsis	Bacterial sepsis fungal meningitis	SLC6A16 SLC6A20	RAB geranylgeranylation	Vindesine Pomalidomide	Modafinil Tiagabine
Asthma exacerbation	Asthma status asthmaticus	NR3C1 NR3C2	Olfactory Signaling Pathway	Prednisolone*^a^ Caffeine*	Betamethasone* Deflazacort*
Dermatologic complications/pressure ulcer	Decubitus ulcer dermatographia	HOXA1 ADNP	tRNA Aminoacylation	Gallium citrate Ga 67 Temazepam	Drostanolone Citric Acid
COPD	Dressler's syndrome obstructive lung disease	NR4A3 KCNA10	Nuclear Receptor transcription pathway	Prednisolone*^a^ Acarbose	Betamethasone Deflazacort
Diabetic ketoacidosis/hyperglycemia ketosis	Type 2 diabetes mellitus diabetic retinopathy	NR4A3 NR3C2	Nuclear Receptor transcription pathway	Prednisolone^a^ Prasterone	Betamethasone Deflazacort
CHF	Congestive heart failure Systolic heart failure	OSBPL8 OSBPL5	Nuclear receptor transcription pathway	Prasterone Trandolapril	Betamethasone Levonorgestrel
Respiratory failure	Respiratory failure	CDIPT SESTD1		Gallium citrate Ga 67 Kanamycin	Citric acid Succinic acid
Pulmonary embolism	Pulmonary embolism and infarction	C5orf52 L3HYPDH	Glutathione conjugation		Gadobutrol Amphetamine
ARDS	Adult respiratory distress syndrome	RETSAT KCNA10	RAB geranylgeranylation	Tetrahydrofolic acid Vindesine	Betamethasone* Levonorgestrel

See Table 1 for details. The top 20 list of comorbidity ranked drugs are in SI, Table S2. *Indicates the drug is currently undergoing clinical trials for the COVID-19 complication in column 1. ^a^Are drugs under trial for a general COVID-19 patient.

### Diseases comorbid with COVID-19

Among the significant comorbidities predicted using the interactome data, there were 250 neurologic, 50 respiratory, 51 hematologic, 33 endocrine, 18 ocular symptoms, 11 renal/acute kidney failure injury, 11 cardiovascular/arrhythmia, 8 sepsis, 7 hepatocellular injury /liver failure, 6 cerebral ischemia/infarction, 5 gastrointestinal symptoms, 5 bacteremia, 3 dermatological, 1 pulmonary embolism, 1 pneumothorax, 1 pneumonia, 1 Disseminated intravascular coagulation (DIC), 1 asthma exacerbation, 1 acute myocardial infarction, and 1 ARDS. From GWAS, there were 101 neurologic, 51 cardiovascular/arrhythmia, 37 endocrine, 31 ocular symptoms, 29 acute hepatitis/liver failure, 16 renal/acute kidney failure injury, 14 cerebral ischemia/infarction, 10 acute myocardial infarction, 8 hematologic, 6 gastrointestinal symptoms, 6 bacteremia, 4 sepsis, 4 asthma exacerbation, 3 dermatological, 3 chronic obstructive pulmonary disease (COPD), 2 diabetic ketoacidosis/hyperglycemia ketosis, 2 congestive heart failure (CHF), 1 respiratory failure, 1 pulmonary embolism, and 1 ARDS complications.

### Pathway analysis

To assess each manifestation’s molecular features, a p-value weighted frequency ranks the comorbidity enriched MOA proteins. This allows for a more expansive list of high confidence putative key proteins and doesn’t solely rely on the input proteins/genes. MOA proteins above score of 0.1 (equivalent to 10% of comorbid indications sharing this MOA protein) (see SI) were used as input into the global pathway analysis for each manifestation. The top pathway from the interactome and GWAS input are shown in Tables 1 and 2, with full lists in Tables S1 and S2. The top 20 most frequent significant pathways across clinical manifestations calculated from the interactome and GWAS inputs are in Tables 3 and 4. Combining this with the hierarchically ranked pathways for each clinical manifestation also allowed us to identify pathways attributed to loss of sense of smell, cytokine storms, blood clots and neurological symptoms.

**Table 3:** Top 20 most frequent pathways across the interactome clinical manifestations.

Pathway|top pathway	Frequency of clinical manifestations
RAB geranylgeranylation|metabolism of proteins	9
Translocation of SLC2A4 (GLUT4) to the plasma membrane|vesicle-mediated transport	4
Transcription of E2F targets under negative control by DREAM complex|cell cycle	4
Sensory processing of sound|sensory perception	4
Sensory processing of sound by outer hair cells of the cochlea|sensory perception	4
Sensory processing of sound by inner hair cells of the cochlea|sensory perception	4
Sema4D in semaphorin signaling|developmental biology	4
Sema4D induced cell migration and growth-cone collapse|developmental biology	4
RHO GTPases activate ROCKs|signal transduction	4
RHO GTPases activate PAKs|signal transduction	4
RHO GTPases activate CIT|signal transduction	4
Regulation of actin dynamics for phagocytic cup formation|immune system	4
Parasite infection|disease	4
Leishmania phagocytosis|disease	4
G0 and early G1|cell cycle	4
FCGR3A-mediated phagocytosis|disease	4
Fcgamma receptor (FCGR) dependent phagocytosis|immune system	4
EPHA-mediated growth cone collapse|developmental biology	4
Nuclear receptor transcription pathway|gene expression (transcription)	3
Kinesins|hemostasis	3

**Table 4:** Top 20 most frequent pathways across the GWAS clinical manifestations.

Pathway|top pathway	Frequency of clinical manifestations
Nuclear receptor transcription pathway|gene expression (transcription)	10
Class A/1 (Rhodopsin-like receptors)|signal transduction	9
Peptide ligand-binding receptors|signal transduction	8
Olfactory signaling pathway|signal transduction	8
Na + /Cl − dependent neurotransmitter transporters|transport of small molecules	7
GPCR ligand binding|signal transduction	7
G alpha (s) signaling events|signal transduction	7
Amine ligand-binding receptors|signal transduction	7
ADORA2B mediated anti-inflammatory cytokines production|disease	7
Tachykinin receptors bind tachykinins|signal transduction	6
Signaling by GPCR|signal transduction	6
Sensory perception|sensory perception	6
GPCR downstream signaling|signal transduction	6
Adrenoceptors|signal transduction	6
PP2A-mediated dephosphorylation of key metabolic factors|metabolism	5
Noncanonical activation of NOTCH3|signal transduction	5
Leishmania parasite growth and survival|disease	5
G alpha (q) signaling events|signal transduction	5
G alpha (i) signaling events|signal transduction	5
Anti-inflammatory response favoring Leishmania parasite infection|disease	5

### Loss of sense of smell

The olfactory signaling pathway^22^ is associated with 8/21 clinical manifestation groups from the GWAS results: acute myocardial infarction, asthma exacerbation, cardiovascular/arrhythmia, COPD, endocrine, hematologic, hepatocellular injury/acute hepatitis/liver failure, and respiratory. There is clinical evidence that some individuals infected with SARS-CoV-2 experience a loss of smell and taste^22^. Note that olfactory receptors may be an alternative SARS-CoV-2 entry into the local host cells, which may lead to its spread into the central nervous system^23^.

### Neurological symptoms

There have been a number of unusual COVID-19-related neurological symptoms such as stroke, confusion, and as previously mentioned, loss of sense of smell and taste^24^. The most frequent clinical manifestation group for the interactome results was neurologic whose top pathway is glutathione conjugation associated with facilitating xenobiotic metabolism. Dysregulation of glutathione plays a role in many diseases including neurodegenerative diseases and cancer^25, 26^. Decreased glutathione levels can lead to oxidative stress, resulting in Parkinson’s and Alzheimer’s disease. Moreover, an imbalance in glutathione levels can impact the immune system^26^. Decreased glutathione concentration is highly associated with serious manifestations causing increased COVID-19 mortality, possibly from increased susceptibility to uncontrolled viral replication^27^.

The most frequent clinical manifestation group from the GWAS results was also neurological. The second top pathway was Na + /Cl− dependent neurotransmitter sodium symporters, which use sodium and chloride electrochemical gradients to import/export several substrates. They are associated with Parkinson’s disease, orthostatic intolerance, and depression^28^. A meta-analysis found that low blood sodium increases the risk and severity of COVID-19^29^. Thus, neurotransmitter transporters that depend on Na + could be dysregulated due to decreased blood sodium levels.

### Blood clotting

Increased blood clots are seen in COVID-19 infected individuals which causes increased mortality^30^. The third most frequent clinical manifestation group for the interactome results was hematologic. RAB geranylgeranylation, the third most frequent pathway across all clinical manifestations from the interactome, is a post-translational modification that allows RABs to connect with intracellular membranes where they regulate vesicle transport pathways^31^. Dysregulation of RAB geranylgeranylation transferase function is linked to abnormal blood clotting.

### Cytokine storms

Several COVID-19 patients face respiratory complications, which may be due to anti-inflammatory cytokines. Using the GWAS input, we find the ADOR2AB mediated anti-inflammatory cytokine production pathway associated with the respiratory manifestation. Over-secretion of interferons can yield uncontrolled systemic inflammation^32^. The ADOR2AB mediated anti-inflammatory cytokine production pathway is involved in 7/21 clinical manifestations. GWAS input also yields the interleukin-1 signaling pathway associated with the CHF clinical manifestation. Interleukin-1 is up-regulated in CHF patients and is a target for treating heart-related diseases^33^.

The second most frequent clinical manifestation group was cardiovascular. One significant pathways is G alpha (s) signaling events, involving 7/21 GWAS clinical manifestation groups. This pathway activates adenylate cyclase producing cAMP. G-protein receptors are associated with heart disease. Among the proteins in this pathway, C5aR1 is a G-protein-coupled receptor. The C5a-C5aR1 complex is involved in COVID-19 progression and is part of a potential therapeutic strategy^34^.This complex is associated with the innate immune response, with C5 a key driver in complement-mediated inflammation^34^.

Examples of immune-related pathways from the interactome clinical manifestation results include regulation of actin dynamics for phagocytic cup formation, the NLRP1 inflammasome, and RUNX3 regulation of immune response and cell migration.

### Uncharacterized manifestations

480 significant comorbidities were not mapped to a known COVID-19 clinical manifestation group from the interactome results. To further understand their effects, we grouped them by their main ICD-10 classification and performed CoPathway analysis. The top three uncharacterized groups were neoplasms, Congenital malformations/deformations/chromosomal abnormalities, and digestive system diseases. The comorbidities, comorbidity enriched MOA proteins and pathways for the interactome results are shown in SI, Table S3.

285 diseases were not mapped to a clinical manifestation group from the GWAS results. Their comorbidities, comorbidity enriched MOA proteins and pathways for the GWAS results are shown in Table S4. The top three uncharacterized groups involve mental and behavioral disorders, diseases of the digestive system, and neoplasms.

### Neoplasms

The interactome input resulted in 90 pathways with a *p*-value < 0.05 involving neoplasms, with many involving hormonal regulation. The top pathway, activation of AMPK downstream of NMDARs, is associated with the neuronal system. AMPK is an enzyme that regulates cellular energy and homeostasis via activating catabolic pathways while switching off cellular growth and proliferation^35^. AMPK has been targeted for cancer treatment because its activation can reduce cancer incidence. NMDARs control synaptic plasticity and memory. Increased expression of NMDARs occurs in a variety of cancers such as neuroblastoma, breast, small-cell lung, and ovarian cancer^36^. Anti-NMDAR encephalitis, characterized by abnormal neurological and behavioral symptoms, has been reported in both COVID-19 and herpes simplex virus 2 (HSV-2)^37, 38^. Notably, HSV-2 can lead to an increased cervical cancer risk^39, 40^.

GWAS provides 29 neoplasm related pathways with a *p*-value < 0.05. The top pathway is the nuclear receptor transcription pathway. Nuclear receptors are DNA-binding transcription factors capable of binding hormones, vitamins, small molecules, and other ligands. A number of underlying disease mechanisms associated with dysregulation of nuclear receptors that can result in cancer, diabetes, and hormone-related conditions. Nuclear receptors have been targeted by cancer therapeutics as they are key players in gene regulatory networks^41^. There has not been substantial research on the relationship between nuclear receptors and COVID-19; but we note that some viruses target nuclear receptors as part of their replication process^42^.

### Is SARS-CoV-2 an oncovirus?

As indicated above, there were many disease comorbidities associated with neoplasms from both the interactome and GWAS results. Neoplasms cause abnormal tissue growth, a significant cancer characteristic. Perhaps, SARS-CoV-2 hijacks the human host replication machinery or proliferation pathways^6^. Indeed, viruses can initiate signal transduction pathways leading to cytokine and chemokine expression. They also dysregulate signaling pathways to promote viral infection and cellular transformations^43^ that elicit a proinflammatory response similar to cancer^44^. A salient example is Human Papillomavirus (HPV). Most cervical cancers^45^ are caused by the cytokine flux associated with inflammation post-HPV infection^40^. Furthermore, the second most significant COVID-19 comorbid disease, T-cell leukemia, is linked to the human T-cell lymphotropic virus (HTLV-I), an RNA retrovirus. More generally, a number of oncoviruses cause cancer^46^. Certain viruses transform human cells causing loss of ability to regulate cell division.

Although we do not yet know the long-term consequences post-COVID-19 infection, these results raise the distressing possibility that SARS-CoV-2 is an oncovirus. To assess this potential relationship, we screened our comorbidity enriched MOA proteins associated with neoplasms from both the interactome and GWAS sets against the COSMIC^47^ database gene set containing 723 oncogenes. There were 1488 and 97 neoplasm comorbidity enriched MOA proteins using the interactome and GWAS as input, respectively (comorbidity weighted frequency > 0.1). From this, 12.3% (n = 89) and 0.97% (n = 7) of the interactome and GWAS comorbidity enriched MOA proteins, respectively, are oncogenes in the COSMIC database(42); see SI Tables S6, S7. The overlap between the neoplasm comorbidity enriched MOA proteins from the interactome as input and the COSMIC database oncogenes resulted in a significant *p*-value of 2.5 × 10^–5^. The overlap between the GWAS neoplasm comorbidity enriched MOA proteins and COSMIC yielded an insignificant *p*-value of 0.08.

As further substantiation of the conjecture that SARS-CoV-2 is an oncovirus, we compared the differential gene expression analysis of COVID-19 patients (n = 1918 differentially expressed genes with an adjusted *p*-value < 0.05), to the COSMIC database^47^. 11% (n = 82) of the genes overlap with oncogenes in the COSMIC database (see SI Table S8). Although the overlap p-value is not significant, this indicates that there are some overlapping oncogenes possibly associated with COVID-19. We next performed a 3-way merge between the interactome/GWAS neoplasm comorbidity enriched MOA proteins, the SARS-CoV-2 differentially expressed genes, and the COSMIC database oncogenes. We found 11 and 1 overlapping gene(s) from the interactome and GWAS 3-way merge, respectively. The pathway analysis on the overlapping COVID-19 differentially expressed genes and the COSMIC database indicates that viral replication and oncogenesis employ similar biochemical mechanisms. Indeed, a number of the identified pathways such as interferon-gamma signaling^48, 49^, immunoregulatory interactions between lymphoid and non-lymphoid cell^50^, and antigen processing-cross presentation^51, 52^ are related to viral replication and oncogenesis. Clearly, additional investigation is needed to explore the possibility that SARS-CoV-2 might be an oncovirus. If this conjecture were true, it would provide an even greater incentive for people to get vaccinated.

### Comparative study of viruses for oncogenic propensity

To further support our conjecture that SARS-CoV-2 might be an oncogenic virus, we compiled the virus-human host interacting proteins of 13 viruses from the literature^53–63^. After applying the same LeMeDISCO procedure as done for SARS-CoV-2 to prioritize these interactomes, we examined the top 100 proteins and their overlap with the 723 COSMIC census putative cancer drivers^47^. Here, LeMeDISCO uses only the top 100 comorbid indications associated with Neoplasms (mapped according to their ICD-10 main codes) to prioritize the MOAs of these Neoplasm indications. We use the p-value (calculated using Fisher’s exact test^64^) of the overlapped proteins as the propensity that ranks the virus’s likelihood of being oncogenic. In practice, if the *p*-value < 0.05, then we consider the virus is oncogenic.

The results are summarized in Table S9. For the 9 known oncoviruses, we failed for 3: Ad5, KSHV and HTLV. However, for the 4 viruses that are not oncoviruses, none has a *p*-value < 0.05. This indicates for these viruses, the false positive rate is 0. SARS-CoV-2 has a *p*-value of 0.016 that is close to those (0.04) of HIV and PyV oncoviruses. Thus, SARS-CoV-2 is closer to oncoviruses than to non-oncoviruses. We should point out that it may take over a decade or longer for cancer to emerge post-infection^65^. Thus, increased cancer rates might be a long term consequence of COVID-19. At present, there is a report lung cancer metastases have increased during the pandemic ^66^ as well as a conjecture that SARS-CoV-2 can induce glioma tumorigenesis^67^.

### Predictions of possible repurposed drug to treat COVID-19’s comorbid diseases

To identify potential repurposed drugs from DrugBank^13^ that might treat a given SARS-CoV-2 complication, MOATAI-VIR utilizes the LeMeDISCO disease profiles for identifying comorbidity-based treatments. For COVID-19’s clinical manifestations, possible treatments are identified by CoMOAdrug and CoVLS. CoMOAdrug identifies drugs that might treat the comorbid diseases to the given disease via indication-based virtual ligand screening using MEDICASCY^12^. A drug is ranked by the fraction of comorbid diseases with that indication weighted by the drug-indication predicted precision inferred from MEDICASCY benchmarking^12^. This yields a rank ordered list, LIST_CoMOAdrug_, for drugs common to the comorbid diseases. CoVLS identifies efficacious drugs by FINDSITE^comb2.0^^68^ virtual ligand screening of the comorbidity frequency weighted MOA proteins of the given adverse response. A drug is ranked by the product of the predicted molecule’s binding precision times the p-value weighted frequency that the protein is a MOA protein of a comorbid disease divided by the summed binding precision of all its human targets. The resulting list is LIST_CoVLS_. A highly ranked drug often has multiple targets with high p-value weighted comorbidity frequency; for additional details, see SI.

### Benchmarking of the LeMeDISCO approach to identify drugs

To prove that comorbidity-based drug ranking works in principle, using MEDICASCY we successfully predicted novel, anti-proliferative small molecules in 79.4% of the top ranked 20 of 1597 molecules from the NCI diversity set in 10 different NCI-60 cancer cell lines^69^. If protein targets are selected based on their p-value weighted comorbidity frequency and binding precision, then the success rate is 85.7% for the top ranked 20 of 1597 molecules.

To further benchmark the ability to map drugs to their predicted indications that also shows the generality of our methodology, we utilized 2,059 training drugs with 123,146 drug-indications pairs in a modified jackknife test. For each drug, when predicting its indications, we used models trained from other drugs having a Tanimoto Coefficient Tc < 0.8 to the given drug. We then evaluated the top 20 drug predictions for each indication using the above two strategies and compared the results to the single indication based MEDICASCY predictions (see Table S5). Since all indications of a given drug are not known, the estimated precision is a lower bound to the true precision^70^. CoVLS has the best lower bound precision of 72.6%, MEDICASCY has the smallest lower bound precision of 58.0%, and CoMOAdrug is in-between, having a lower bound precision of 64.2%. The mean enrichment factors within the top 20 by CoMOAdrug and CoVLS are 7.05 and 7.97, respectively. Thus, CoMOAdrug and CoVLS show significant enhancement in their ability to select efficacious drugs over random.

### Potential candidate repurposed drugs to treat COVID-19 complications

Tables 1 and 2 also present the top 2 predicted repurposed drugs to treat COVID-19’s major adverse complications using both CoMOAdrug and CoVLS ligand ranking approaches based on the SARS-CoV-2 human interactome and GWAS, respectively. The full list of high-ranking repurposed drugs for the 24 mapped complications for inputs of human interactome and GWAS risk genes are given in Tables S1 and S2, respectively. Drugs selected by CoMOAdrug and CoVLS drugs have a similar selection frequency. For the top 20 drugs, CoMOAdrug selects 53/139 (38%) drugs for only one complication, while CoVLS selects 73/199 (37%) drugs that appear in just one complication.

Next, ClinicalTrials.gov data (data obtained October 2020) was mined to determine how many of the drugs predicted for the COVID-19 complications are currently undergoing clinical trials for their respective COVID-19 complication. For the 24 predicted complications, the average number of FDA-approved drugs undergoing clinical trials is 4.1 (~ 21%) within the top 20 predicted drugs (4.3 for CoMOAdrug and 3.9 for CoVLS). In a sense, this is a soft validation of MOATAI-VIR. Drugs undergoing clinical trials for their respective COVID-19 complication are marked with “*” in SI Tables S1 and S2. Furthermore, drugs undergoing clinical trials for generic treatment of COVID-19 are marked with “$” in SI Tables S1 and S2.

To further establish the plausibility of the drug predictions, a literature search of several drugs from the comorbidity-associated complications was done. For example, Prednisolone, typically used for treating allergies and infections^71^, is the top drug predicted using CoMOAdrug for the respiratory clinical manifestation from the interactome results. Prednisolone is a glucocorticoid with anti-inflammatory, immunosuppressive, anti-neoplastic, and vasoconstrictive MOA. Corticosteroids reduce adverse events attributed to ARDS^72^. Prednisolone was also the top drug for the asthma exacerbation clinical manifestation from GWAS, whose top pathway was olfactory signaling. MEDICASCY predicts that Prednisolone has 142 indications which overlap 13 of COVID-19’s complications or their comorbid indications.

The top drug predicted using CoVLS for the respiratory clinical manifestation from GWAS was Betamethasone, another corticosteroid with immunosuppressive and anti-inflammatory properties. Vindesine, an inhibitor of mitosis and a chemotherapeutic^73^, was among the top 5 drugs found from both the CoVLS and CoMOAdrug methods for the interactome hematological clinical manifestation. Vindesine can lower the number of platelets in the blood, thus, preventing blood clots^74^.

The immunomodulatory drug, Pomalidomide, an FDA-approved thalidomide derivative for treating multiple myeloma^75^ was among the top predicted drugs by CoMOAdrug for the neurologic clinical manifestation from the interactome input. Pomalidomide has been investigated for use in neurological conditions such as Parkinson’s disease. It can improve age-related neurological impairment/motor disability^76^ and can reduce ischemic brain injury in an in vivo study^77^.

Temazepam was in the top 20 predicted drugs using the CoMOAdrug method for acute myocardial infarction/unstable angina, hematologic, neurologic, and asthma exacerbation clinical manifestations from the GWAS results. Temazepam is used to treat insomnia and is suggested as a treatment for sleep-related disturbances associated with COVID-19^78^.

### Systematic validation of predicted drugs to treat COVID-19 complications

The above results are promising. However, drugs in clinical trials might not work in practice. Nevertheless, to have an idea of how well our method can do compared to similar drug prediction methods as in^79^ for predicting drugs that directly treat COVID-19, we performed a similar assessment by using the clinical trial drugs as true positives and others as true negatives to calculate the area under the ROC curve (AUROC). For the interactome input, the mean AUROCs of CoMOAdrug and CoVLS are 0.70 and 0.73, respectively. For the GWAS input, they are 0.71 and 0.73, respectively. These are comparable to the non-AI-based methods of^79^. Our method does not have any AI-based method trained using SARS-CoV-2 related information whereas AI-net of^79^ does.

To systematically validate our predictions, we utilized our training drug-indication dataset in MEDICASCY that was curated by experts. Since all predictions are carried out by training models on drugs having Tc < 1 to the drug whose efficacy is predicted, they are true predictions and not just memorization of known indication-drug relationships. We use an enrichment factor within the top 20 of the total 2095 screened drugs (~ top 1%). Since the AUROC depends on true positives in the middle of the ranking, we instead calculate the AUPRC (area under precision-recall curve) which depends on true positives ranked at the very top. The results are shown in Table 5. The mean enrichment factors of CoMOAdrug and CoVLS drugs for interactome input are 3.91 and 14.6, respectively. For GWAS input, they are 3.36 and 7.99, respectively. These are consistent with a benchmarking test (Table S5) and are far better than random selection. The mean AUPRCs (around 0.09 to 0.184) are consistent with an earlier MEDICASCY benchmark^12^ and better than those of the indication prediction method of Himmelstein et al*.*^80^ (~ 0.005 to 0.1) where a systematic integration of biomedical knowledge was used for computing drug features and a logistic regression machine learning was employed for learning and prediction.

**Table 5:** Enrichment factor within top 20 and AUPRC of predicted drugs to treat COVID-19 complications.

Complication	Interactome		GWAS	
	CoMOAdrug	CoVLS	CoMOAdrug	CoVLS
Respiratory	5.19/0.097	3.11/0.100	3.41/0.249	2.73/0.282
Pneumonia	15.0/0.218	15.0/0.047	NA	NA
Respiratory failure	0/0.003	34.9/0.174	4.55/0.042	6.83/0.062
Acute respiratory distress syndrome (ARDS)	2.44/0.046	4.87/0.111	2.44/0.046	4.87/0.111
Asthma exacerbation	0/0.002	26.2/0.131	6.55/0.140	7.37/0.215
Pneumothorax	0/0.003	34.9/0.174	NA	NA
Chronic obstructive pulmonary disease (COPD) exacerbation/acute coronary syndromes	NA	NA	6.79/0.134	3.88/0.173
Cardiovascular/arrhythmia	2.10/0.037	4.19/0.046	3.15/0.372	2.73/0.398
Acute myocardial infarction/unstable angina	8.06/0.054	8.06/0.030	3.71/0.232	1.85/0.176
Acute congestive heart failure (CHF)	NA	NA	6.12/0.229	1.36/0.159
Hematologic	3.68/0.267	4.02/0.285	3.27/0.117	4.91/0.135
Pulmonary embolism	NA	105/0.083	NA	105/0.083
Disseminated intravascular coagulation (DIC)	0.0/0.0	0.0/0.020	NA	NA
Neurologic	2.44/0.202	2.03/0.211	2.94/0.339	3.43/0.377
Cerebral ischemia/infarction	0.0/0.014	5.82/0.025	2.79/0.156	2.79/0.173
M Endocrine	4.71/0.195	4.12/0.169	2.62/0.288	1.83/0.304
Diabetic ketoacidosis/hyperglycemia and ketosis	NA	NA	3.31/0.173	2.76/0.158
Gastrointestinal symptoms	3.61/0.057	10.8/0.098	1.97/0.170	3.93/0.228
Hepatocellular injury/acute hepatitis/liver failure	3.64/0.113	2.91/0.110	1.99/0.268	1.42/0.242
Renal/acute kidney failure or injury	3.27/0.172	1.64/0.155	0.576/0.135	3.45/0.162
Sepsis	4.76/0.104	10.7/0.167	0.0/0.013	0.0/0.011
Bacteremia	3.61/0.057	10.8/0.098	1.97/0.170	3.93/0.228
Dermatologic complications/pressure ulcer	8.06/0.071	6.04/0.065	6.16/0.015	0.0/0.027
Ocular symptoms	7.62/0.096	11.4/0.150	2.91/0.114	2.91/0.153
Mean	3.91/0.090	14.6/0.117	3.36/0.170	7.99/0.184

The first number is the enrichment factor and the second is the AUPRC.

### Predicted repurposed drugs to treat loss of sense of smell

Using CoMOAdrug and CoVLS, we predicted repurposed, FDA-approved drugs that possibly target the olfactory signaling pathway as prospective treatments for loss of the sense of smell. The top 20 drugs provided by each approach are shown in Table 6 Theophylline, a drug known to treat respiratory diseases, such as COPD and asthma was predicted by **CoVLS** and is currently undergoing clinical trials for anosmia (loss of sense of smell)^78^.

**Table 6:** Top 20 drugs predicted to target the olfactory signaling pathway for treating the highlighted symptom, loss of sense of smell.

CoMOAdrug drugs	CoVLS drugs
Cabazitaxel	Fluorouracil
Prasterone	Imiquimod
Lactose	Pamabrom
Boldenone	Fenethylline
Paclitaxel	Dimenhydrinate
Vinorelbine	Theobromine
Idarubicin	Caffeine
Vincristine	Theophylline*
Ifosfamide	Aminophylline
Trifluridine	Oxtriphylline
Tibolone	Nevirapine
Icotinib	Ethynodiol diacetate
Triptorelin	Cilostazol
Cyclophosphamide	Vapreotide
Etonogestrel	Clonazepam
Gonadorelin	Desogestrel
Hexaminolevulinate	Enprofylline
Betazole	Flunitrazepam
Cisplatin	Linagliptin
Mitoxantrone	Mefloquine

*Indicates the drug is currently undergoing clinical trials for loss of smell.

## Discussion

MOATAI-VIR can identify possible molecular mechanisms responsible for COVID-19’s severe adverse consequences. Not only are most of COVID-19’s severe symptoms successfully predicted, but Tables 1 and 2 suggest a list of possible repurposed and mostly untested drug treatments for these complications. They could also be combined with antiviral drugs that directly target SARS-CoV-2 proteins to kill the virus. The goal is to mitigate both COVID-19 infection and its subsequent adverse complications to improve clinical outcome. Thus, MOATAI-VIR provides a series of logical, systematic suggested treatments for COVID-19’s adverse reactions. Equally important, MOATAI-VIR is a general methodology for antiviral drug repurposing that can be applied to new outbreaks of other novel viral infections as they emerge.

One possible limitation of the current method as with all in silico drug predictions is that the predictions are not 100% accurate. Even though we have an enrichment factor much better than random, there are still many false positives. Further studies that could improve drug predictions that we will undertake in the near future are as follows: For MEDICASCY, we plan to include the more accurate protein structure models from AlphaFold 2^81^. Then, to improve its ligand virtual screening component, we shall employ the better quality structure models of AlphaFold 2 and the better screening method of FRAGSITE^82^ as well as its consensus ligand binding proteins identified in combination with FINDSITE^comb2.0^^68^. Another limitation is that the current method is population based, i.e., comorbidities and drugs are predicted for the whole population, not specific individuals. For specific individuals, some comorbidities may not occur, and the predicted drugs may not work. These problems could be addressed by including personal genetic information and gene expression profiles of the appropriate tissues (e.g. the lungs) of SARS-CoV-2 patients to identify the specific proteins in that that are affected by SARS-CoV-2.

## Materials and methods

Here, we give a brief description of the methods used in MOATAI-VIR, with additional details provided in SI. We start by performing large-scale prediction of MOAs for 3,608 indications using MEDICASCY^12^. MEDICASCY predicts MOAs for given indication by combining its drug-indication predictions and drug whole human genome protein target predictions from FINDSITE^comb2.0^^68^. A p-value is derived for each indication-human protein pair using Fisher’s exact test^64^. Using a *p*-value cutoff of 0.05, for each indication, we define a list of putative MOAs. To eliminate false positive predictions, the human atlas protein expression data is utilized to exclude proteins “not detected” in relevant tissues of a given indication^83^. Next, we apply LeMeDISCO (see SI for details) to examine the overlap proteins of the input interactome and GWAS sets to the MOA proteins of each of the 3,608 library indications to obtain their co-morbid indications. LeMeDISCO calculates a Jaccard index for ranking and the corresponding p-value using Fisher’s exact test to determine the comorbidity of two sets of proteins. With a p-value cutoff of 0.05 for comorbidity, we obtained comorbid indications for the interactome and GWAS inputs. Then, comorbid indications are mapped to their respective COVID-19 complications. We subsequently employ CoPathway to determine significant pathways associated with the most frequent comorbidity enriched MOA proteins. We assess the frequency of MOA proteins across the comorbidities for a desired group and then processes the top ranked (comorbidity ranked *p*-value weighted frequency > 0.1) MOA proteins through the Reactome^84^ for global pathway analysis. Pathways with a *p*-value < 0.05 are deemed significant_._ Lastly, two methods, CoMOAdrug and CoVLS, for drug discovery of the mapped indications were performed. CoMOAdrug screens FDA-approved drugs by combining the screening from MEDICASCY on the top 100 comorbid indications of a given indication. CoVLS screens drugs for efficacy by screening all comorbid frequency ranked MOAs of a given indication against FDA-approved drugs using FINDSITE^comb2.0^.

## Supplementary Information

Supplementary Information 1.

Supplementary Information 2.

## Data Availability

In addition to the Supplementary information associated with this paper, all Supplementary Tables are available on our website at https://sites.gatech.edu/cssb/moatai-vir/https://sites.gatech.edu/cssb/moatai-vir/.
