# Supplementary material for: Prediction of severe adverse events, modes of action and drug treatments for COVID-19’s complications
Source: Sci Rep. 2021 Oct 21;11:20864. doi: 10.1038/s41598-021-00368-6 (PMC8531388; doi:10.1038/s41598-021-00368-6)
Supplement: Supplementary file 2 — Supplementary Information 2. [file 41598_2021_368_MOESM2_ESM.docx]

**Supplementary Information for**

Prediction of severe adverse events, modes of action and drug treatments for COVID-19’s complications

Courtney Astore, Hongyi Zhou, Joshy Jacob, Jeffrey Skolnick

Jeffrey Skolnick

Email: [skolnick@gatech.edu](mailto:skolnick@gatech.edu)

**This PDF file includes:**

Supplementary text

Figures S1 to S4

Tables S0 to S9

SI References

Supplementary Information Text

**Supplementary Materials and Methods**

An overview of the **MOATAI-VIR** approach is shown in the main text Figure 1, with a detailed flowchart shown in Figure S1. The goal of **MOATAI-VIR** is to identify severe adverse responses associated with SARS-CoV-2 and their corresponding human mode-of-action (MOA) proteins. This information is then used to predict possible FDA-approved drugs that could alleviate these dangerous responses. To accomplish these objectives, we input either the experimentally determined human proteins from the human-SARS-CoV-2 interactome^1^ or the COVID-19 GWAS survival associated risk genes^2,3^ as a set of MOA proteins in a SARS-CoV-2 MOA profile. We then employ our recently developed **LeMeDISCO** algorithm which compares these SARS-CoV-2 MOA proteins to the **MEDICASCY**^4^ predicted MOAs of 3,608 diverse disease indications. **LeMeDISCO** calculates a J-score^5^ whose associated p-value^6^ determines significantly associated diseases with either the human component of the SARS-CoV-2-human interactome or the GWAS-based SARS-CoV-2 MOA protein profiles^1-3^. **LeMeDISCO** then outputs indications that likely cause the adverse events resulting from SARS-CoV-2 infection.

To identify repurposed drugs that might treat a given SARS-CoV-2 secondary effect/symptom (comorbid diseases), **MOATAI-VIR** utilizes the **LeMeDISCO** disease profiles for identifying comorbidity-based treatments. For COVID-19’s secondary effects, possible treatments are identified by two complementary approaches, **CoMOAdrug** and **CoVLS. CoMOAdrug** identifies drugs to treat the comorbid diseases to the given disease via indication-based virtual ligand screening using **MEDICASCY**^4^. A drug is ranked by the fraction of its comorbid diseases with that indication weighted by the drug-indication’s predicted precision. A predicted precision is inferred from **MEDICASCY**^4^ benchmark testing (Figure S2). This yields a rank ordered list, LIST_CoMOAdrug_, for drugs common to the comorbid diseases. **CoVLS** identifies efficacious drugs by **FINDSITE^comb2.0^**^7^ virtual ligand screening (VLS) of the comorbidity frequency weighted MOA proteins of the given adverse response. Here, a drug is ranked by the summed score of all its MOA targets. The score for a given target is defined as product of the predicted molecule’s binding precision times the -log p-value weighted frequency (see below) that the protein is a MOA of a comorbid disease divided by the summed binding precision of all its human targets. The resulting list is LIST_CoVLS_. A highly ranked drug often has multiple targets with high comorbidity frequency. All screened drugs are repurposed FDA-approved drugs from DrugBank^8^. The goal is to provide distinct, targeted possibly ready-to-use therapeutics based on the adverse secondary effect attributed to SARS-CoV-2 infection. We detail each of these steps in what follows.

**Application of MEDICASCY for the large-scale prediction of MOA proteins for diseases**

While **MEDICASCY** has been published^4^, here, a brief description is provided for the convenience of the reader. The flowchart in Figure S2 shows how indications are predicted by **MEDICASCY**. **MEDICASCY** requires the structures of all proteins in the human exome. These were modeled by a fast version of the **TASSER^VMT^** algorithm^9-11^ applied to the entire human exome (<ftp://ftp.ncbi.nih.gov/genomes/H_sapiens/protein/)>. Then, two classes of features were computed for the machine learning of indications from the input drugs: (a) A 256 dimensional molecular fingerprint computed directly from the chemical structure that is converted to its MACCS fingerprint^12^ using Open Babel software (<http://openbabel.org/wiki/Main_Page)>. (b) A feature generated from a drug’s predicted human protein targets using the latest version of **FINDSITE^comb2.0^**’s^7^ virtual ligand screening of the given drug against the 32,584 (97%) human proteins with pre-computed structures and ligand binding pockets^11^. Protein targets are then filtered for possible disease associations by requiring that at least >5% of all possible missense or frameshift/stop mutations of a given protein are disease associated as assessed by **ENTPRISE/ENTPRISE-X**^13,14^. The idea is that if essentially all variations of the protein are neutral, then it is highly unlikely that it is a mode-of-action protein for any disease. **ENTPRISE/ENTPRISE-X** both have low false positive rates, which is essential considering the very large number of variations found in the human exome.

Having predicted that a given protein is associated with some disease, the next issue is to identify which disease. To accomplish this, each protein is mapped to a 960 dimensional disease profile that serves as input to **Know-GENE**^15^, whose output predicts the diseases a protein could be associated with. In principle, these two steps could provide the mapping of proteins to indications. However, this mapping is limited to proteins interacting with those proteins having some (but not all) disease associations that are used for gene-disease association inference.

Next, we employ a more powerful approach that uses drugs as a probe to identify proteins associated with a given disease. To accomplish this objective, a newly developed, Boosted Random Forest (BRF) machine learning approach for multiple label regression is employed for learning and prediction^4^. BRFs build on the Random Forest (RF) approach which is an ensemble learning method for classification and regression^16^. The BRF gives identical results as the RF but is much faster with much smaller memory requirements. The BRF learns indications of a drug by its fingerprint feature from drugs of similar structure and by the disease profile feature from drugs of similar disease profiles derived from their human protein targets. These two features are independently used in both training and prediction. The final prediction score is the average of these two disparate prediction approaches.

In practice, **MEDICASCY** covers 3,608 indications defined in the Human Disease Ontology database^17^. Those indications are collected from three different sources: (1) the approved drug subset from the Therapeutic Target Database (TTD version Sept 12, 2017)^18^, (2) the SIDER4 indication set^19^, and (3) all the clinical trial drug sets were collected from ClinicalTrials.gov and mapped to DrugBank^8^ by Himmelstein *et al*^20^. All disease indications are converted to Human Disease Ontology IDs^17^ (releases/2018-12-17) and merged if the Tanimoto Coefficients (T_c_)^21^ of the two drugs are one. The merged dataset has 2,059 drugs with 3,608 unique indication terms and 123,146 drug-indication pairs. These indications cover diverse disease classes.

**MEDICASCY** can also infer possible MOA proteins for a given indication. Figure S3 shows the flowchart for inferring MOA proteins for a given disease. **MEDICASCY** starts by mapping the probe drugs to their respective indications. Once the drug-protein mapping (as predicted by **FINDSITE^comb2.0^** VLS^7^) is known, we can infer protein-indication mapping. The goal is to infer the putative MOAs of a disease by calculating the enrichment of a protein target T for a given disease D. To achieve this, we use a set of drugs with possible diverse indications as probes to map their indications, and then, by combining this information with **FINDSITE^comb2.0^**’s predicted drug-protein mapping, we infer protein-indication relationships. Here, we choose the 2,095 FDA-approved drugs from the DrugBank (version 5.09) database^8^ as the probe set.

**MEDICASCY** is applied in prediction mode (i.e., any training drugs having a Tanimoto-Coefficient =1 to a given input drug are excluded from training) to avoid a strong bias towards drugs in the training set. For each of the 3,608 indications, we rank the 2,095 probe drugs according to their Z-scores, Z_d_, defined using the raw score computed by **MEDICASCY** from:

$Z_{d}=(\frac{raw score - average raw score of 2,095 drugs}{standard deviation of 2095 raw scores} )$ (1)

To predict a drug as having the given indication, we applied a Z_d_ cutoff of 1.65 that approximately corresponds to a p-value of 0.05 for the upper tailed null hypotheses assuming random raw scores satisfying a normal distribution. We note that this is a different problem than the calculation of the MOA p-value (see below for relative risk) or the comorbidity p-value of overlapped proteins. For the latter two cases, the normal distribution does not always hold. Here, we assume that the random machine learning score is a normal distribution. Thus, for each indication D, the 2,095 probe drugs are separated into two groups: N_1_ are predicted to have indication D (Z_d_$\geq$1.65) and N_2_ (=2,095-N_1_) are not predicted to have indication D (Z_d_<1.65). We should point out that this is a very loose prediction of a drug’s indication. The advantage of using a Z_d_ cutoff for the prediction is that for a given indication, it always predicts some drugs having the indication with its expected statistical confidence.

We then examine each protein target, T, in the human proteome of our modeled 32,584 proteins. There are a subset of the drugs (or perhaps none) predicted by **FINDSITE^comb2.0^**^7^ to bind T. For a given indication D and a human protein target T, we define the relative risk RR(D,T) of the given target T with respect to indication D as:

RR(D,T)=$\frac{fraction of drugs with indication D binding to the target T}{fraction of drugs without indication D binding to the target T}$ ` (2a)

The numerator is the estimation of the probability of drugs having the predicted indication D (Z_d_$\geq$1.65) that bind to protein T and is calculated as F_1_=($N_{1}^{T}$ = # of drugs having Z_d_$\geq$1.65 and binding to T)/N_1_. The denominator is the probability of finding drugs that do not have the predicted indication D but which bind to protein T calculated as F_2_=($N_{2}^{T}$ = # of drugs having Z _d_$<$1.65 and binding to T)/N_2_. This latter probability serves as the background probability that an arbitrary drug will bind to T. When no drug is predicted to bind to protein T, RR(D,T) is set to zero. RR(D,T)=F_1_/F_2_ > 1 means that a drug having indication D is more likely to bind to T than arbitrary drugs not having the predicted indication D will bind to T.

We then compute the statistical significance of RR(D,T) by calculating a p-value using Fisher’s exact test ^6,22^ on the following contingency table for all $N_{1}^{T}$ $\geq$ observed $N_{1}^{T}.$

$\left( \begin{matrix} N_{1}^{T} & N_{1}-N_{1}^{T} \\ N_{2}^{T} & N_{2}-N_{2}^{T} \end{matrix} \right)$ (2b) .

We define a protein target T as predicted to be a possible MOA target for indication D if its p-value<0.05 because it is more likely to be targeted by its efficacious drugs than an arbitrary drug. In the end, for each of the 3,608 indications, there is a list of predicted possible MOA proteins.

To further reduce false positive MOAs, we utilized the human protein atlas database (<https://www.proteinatlas.org/about/download>, *normal_tissue.tsv*) of expression profiles for proteins in normal human tissues based on immunohistochemisty using tissue micro arrays^23^ to remove those proteins that are “not detected” and not “uncertain” in all tested tissues and cell types related to a given indication. To determine which tissues are related to a given indication, tissues are mapped to their ICD-10 main codes. Indications having the same main codes are assumed to involve the same tissue.

**Comorbidity predictions by LeMeDISCO**

A flowchart of the **LeMeDISCO** comorbidity prediction algorithm is shown in Figure S4. Using the input of two sets of putative MOA proteins from two diseases predicted by **MEDICASCY** or obtained from experimental data, one then calculates their Jaccard index^5^ J(D_1_,D_2_) (J-score) and its associated p-value using Fisher’s exact test^6^ for the contingency table in eq. 3.

J-score= $N_{s}$ /(N_D1_+N_D2_$-N_{s}$) (3a)

$\left( \begin{matrix} N_{s} & N_{D2}-N_{s} \\ N_{D1} & N_{t}-N_{D1} \end{matrix} \right)$ (3b)

$N_{D1}$, $N_{D2}$ are the numbers of MOA proteins/genes of disease D_1_, D_2_$; N_{s}$ is the number of overlapped MOAs between D_1_, D_2_ and $N_{t}$ is the total human proteins/genes. The Jaccard index^5^, J-score is a statistical measure of the similarity between MOAs of D_1_ and D_2_. Its value is between 0 and 1. Since the null hypothesis of $N_{s}$ follows a hypergeometric distribution, the p-value for observing the number of overlapped MOA proteins between D_1_, D_2_ $\geq$ $N_{s}$ can be calculated using Fisher’s exact test provided in the contingency table of eq. 3b for all $N_{s}$ $\geq$ observed $N_{s}$ ^22^. We will use the J-score for predicting comorbidity and compare it with the observed comorbidity.

Common probable MOA targets between disease pairs can be derived from either experimental data or **MEDICASCY** predictions. The types of experimental data that can be used include, but are not limited to, differential gene expression (GE), Mendelian or somatic mutation profiles comparing disease vs. control normal samples, better vs. worse prognosis samples, or drug treated vs. control untreated samples^24^. Similarly, the types of input MOA proteins for **LeMeDISCO** are flexible. Thus, **LeMeDISCO** has the unique advantage of being able to test diverse hypotheses relevant to MOA driver protein prioritization, drug repurposing and discovery. It also can prioritize probable MOA proteins for subsequent experimental target validation and has application to precision medicine or targeted therapies. For COVID-19, we use its human interacting proteins^1^ and GWAS risk genes^2,3^ as input MOA protein sets.

**Validating the use of the J-score for comorbidity prediction**

First, we validated **LeMeDISCO**’s J-score in eq. 3 by correlating it with the observed comorbidity as quantified by these two measures: (a) the logarithm of relative risk log(RR) score and (b) the φ**-**score (Pearson's correlation for binary variables)^25^. The relative risk (RR) score is the probability that two diseases occur in a single individual relative to random occurrence and scales exponentially with respect to the strength of two diseases interacting or influencing each other. Thus, instead of using the RR score, as done in previous studies^25-28^, here we use the log(RR) score for correlation analysis. The log(RR) and φ**-**score are computed from US Medicare insurance claim data of approximately 13,038,014 individuals, who had the 32,341,347 inpatient hospital visits^25^ using the following equations:

Log(RR) = log($\frac{n_{AB}/n_{tot}}{{(n}_{A}/n_{tot}){(n}_{B}/n_{tot})})$ (4a)

$\varphi\mathbf{-}score=(n_{AB}*n_{tot}-n_{A}*n_{B})/\sqrt{n_{A}*n_{B}*\left( n_{tot}-n_{A} \right)*\left( n_{tot}-n_{B} \right)}$ (4b)

where n_tot_ = total number of patients in the data set; n_A_, n_B_ = number of patients diagnosed with disease A and B, respectively; n_AB_ = number of patients diagnosed with both diseases A and B.

We also compare the J-score to other scores such as (A) the XD score from known disease-gene associations and protein-protein network propagation^26^, (B) NG, is the number of shared genes between disease pairs^26^, (C) the *S_AB_* score, a protein-protein network-based separation of a disease pair calculated from known disease-gene associations, defined as S_AB_=<d_AB_>-(<d_AA_>+<d_BB_>)/2, where S_AB_ compares the shortest distances between proteins within each disease A & B^28^, <d_AA_> and <d_BB_>, to the shortest distances <d_AB_> between A-B protein pairs^28^, and (D) the symptom similarity score obtained from text mining^27^.

In addition to the correlation analysis, we also calculate the recall rate of each prediction score. This is important in that conclusions drawn from a small fraction of true comorbid diseases might not be true in general. We define a positive comorbidity pair when their log(RR) > 0 or RR>1 and a comorbidity prediction by J-score having a corresponding p-value<0.05, XD score > 0, *S_AB_* score < 0, or the symptom similarity score > 0.1^21^. The recall rate is defined as

$recall=\frac{Number of correctly predicted comorbidity pairs}{Total number of true comorbidity pairs}$ (5)

Table S0 summarizes the results of testing **LeMeDISCO**’s J-score and the comparison to the XD score^26^, NG^26^, the *S_AB_* score^28^ and the symptom similarity^27^ for correlations with comorbidity quantified by the log(RR) score and φ**-**score. Note that the J-score, XD score, NG and symptom similarity are expected to have positive correlations and the *S_AB_* score (a distance measure on interactome between two diseases) is expected to have negative correlations with the log(RR) score and φ**-**score. If the results have opposite correlations, no p-values are provided. Here, we consider the symptom similarity score of ^27^ as a score for predicting comorbidity rather than a measure of likely comorbidity since it is not computed directly from the observed disease frequencies.

Mapping the DOIDs from the Human Disease Ontology database to the ICD-9 IDs of ^25^, we obtain 198,149 disease pairs for use in large scale testing of **LeMeDISCO**. To compare **LeMeDISCO**’s results using J-score with the XD score, which is the closest to our shared MOA protein approach, we mapped their ICD-9 disease code to the DOIDs and obtained a subset of 29,783 pairs from their dataset of 97,665 pairs^26^. For comparison to the *S_AB_* score^28^, the MeSH^29^ disease names^30^ were mapped to DOIDs for consistency. A consensus set of 947 disease pairs from their dataset and our dataset of 198,149 was obtained. A similar dataset of 2,630 disease pairs was obtained for comparison with the symptom similarity score (consensus of Supplementary dataset 4 of ^27^ with the above set of 198,149 pairs). In addition to calculating the Pearson’s correlation of all data points, we also divided the data points into 10 bins according to the respective score range of J-score, XD, *S_AB_* and symptom similarity score. The corresponding log(RR) and φ**-**scores are averaged in each bin. For example, if *S_AB_* is in the range *S_AB_min_* to *S_AB_max_*, a bin size of (*S_AB_max_* - *S_AB_min_*)/10 is used to partition the data points (disease pairs) into 10 bins according to each datum point’s *S_AB_* value. Inside each bin, the log(RR) or φ**-**scores are averaged over the data points; then, the center value of the bin represents the prediction score *S_AB_*. This gives equal weight to the rare prediction scores in the correlation analysis.

For the large set of 198,149 disease pairs, all correlations of J-score with log(RR) score and φ**-**score are statistically significant (p-value < 0.05). We also tested the correlations of the number of shared MOA proteins (NP) between two diseases by **LeMeDISCO**. They are all worse than **LeMeDISCO**’s J-score and have an insignificant (p-value > 0.05) when data binning is applied. For the 29,783 disease pairs used for the XD score comparison, **LeMeDISCO**’s J-score is obviously better than the XD score. Their NG score essentially has no significant correlation with log(RR) and only shows correlation with the φ**-**score for unbinned data. When the data are binned, both the XD score and NG score have no significant correlations. This demonstrates that the shared number of genes derived from known disease-gene associations is not good enough for interpreting comorbidity.

Compared to the *S_AB_* score^28^ on the 947 disease pairs, **LeMeDISCO**’s J-score is better than the *S_AB_* score. The *S_AB_* score has no significant correlation for binned φ**-**score data. The reason for the difference between unbinned and binned data is that the correlation for unbinned data is dominated by those data points where the prediction scores are concentrated. For *S_AB_* scores, 874/947 are > 0, and thus, *S_AB_* score’s correlation for unbinned data is dominated by those points. In this region, no comorbidity is predicted, whereas for binned data, the rare predictions with *S_AB_* score < 0 (where comorbidity is predicted) will have equal effects as those whose *S_AB_* score > 0 on the correlation. We note that the sparse predictions of *S_AB_* score < 0 means that its recall rate is very low if a *S_AB_* score < 0 is a positive comorbidity prediction.

The symptom similarity score has a better correlation than **LeMeDISCO**. However, it only explains the relationship of one phenotype (symptom) to another phenotype (disease) and lacks the ability to identify the underlying molecular mechanism responsible for this comorbidity. The recall for the symptom similarity score is 100% because it already discards non-significant scores (< 0.1). Nevertheless, all correlations of the J-score are statistically significant, and its recall rate is close to 70% for these 2,630 disease pairs. The advantage of the J-score over the symptom similarity score is that it has clear molecular interpretations as it provides the overlapping MOA proteins. Moreover, **LeMeDISCO** does not rely on prior knowledge or symptomatic information for each disease. Hence, it provides a much larger coverage of comorbidity predictions of the 198,149 disease pairs each ranked by its J-score and the corresponding p-value to reflect the expected statistical confidence.

**Predicting COVID-19’s clinical manifestations**

We first predict the severe adverse consequences of COVID-19 using **LeMeDISCO**. **LeMeDISCO** predicts comorbid diseases by scanning the putative MOAs of input disease against those of the 3,608 library diseases. The input MOAs of COVID-19 are from either the experimentally determined human-SARS-CoV-2 interactome^1^ or COVID-19 GWAS survival associated risk genes^2,3^. **LeMeDISCO** then calculates the J-score and corresponding p-value of the input proteins to the MOAs of the 3,608 library diseases taken from **MEDICASCY’s** predictions using eq. 3. Diseases are ranked by their respective J-scores and are predicted as severe adverse consequences of COVID-19 when the corresponding p-value < 0.05.

**Mapping the COVID-19 severe adverse events to clinical/uncharacterized manifestation groups**

The clinical manifestations were first mapped to their corresponding ICD-10 code(s)^31^. Then, the predicted COVID-19 severe adverse events were placed in their corresponding clinical manifestation group via overlapping ICD-10 code(s)^31^. Some severe adverse events fell into multiple clinical manifestation groups as they may have more than one ICD-10 code^31^. To further analyze the predicted comorbidities that did not map to a clinical manifestation group, the remaining diseases were grouped by their main ICD-10^31^ classification for the subsequent analysis.

**CoPathway**

The objective of the **CoPathway** method is to establish lists of significant pathways associated with each clinical/uncharacterized manifestation. The p-value weighted frequency as employed in CoVLS was used to determine which MOA proteins are used for global pathway analysis. Using high-ranking comorbid proteins can identify more relevant proteins in the input interactome or GWAS set. A cutoff of 0.1 (equivalent to 10% of the comorbid indications having MOA proteins with a significant p-value, this cutoff is empirical) for the p-value weighted frequency was used to determine the comorbid enriched MOA proteins to use in global pathway analysis. The Reactome^32^ global pathway analysis tool was used to determine the most enriched and significant pathways associated with the inputted set of proteins for each manifestation group. We then extract the pathways with a p-value < 0.05 and further mapped the pathways to their top pathway classification for further insights and prospective.

**Drug repurposing for the COVID-19 clinical manifestations**

Having discovered a list of severe secondary effects predicted by **LeMeDISCO** and their respective MOAs using the human interactome^1^ or GWAS risk genes^2^, the next step is to find repurposed FDA-approved drugs for possible use in patients exhibiting specific severe secondary effects resulting from SARS-CoV-2 infection. For each Clinical manifestation, we employ **LeMeDISCO** to provide its comorbid diseases and prioritize MOA proteins taken from **MEDICASCY’s** predictions. We selected the top 100 ranked comorbid diseases for further MOA prioritization and drug discovery purposes. For each input protein, we calculate its p-value weighted frequency of being a MOA protein of the comorbid diseases of the given severe adverse consequence. This weighted frequency is then used to rank the MOA proteins for each severe adverse consequence. Again, 2,095 FDA-approved drugs from DrugBank^8^ (v.5.09) are screened for repurposing. Drugs are selected using the following two methods:

**CoMOAdrug**

This method identifies drugs predicted by **MEDICASCY** to be possibly efficacious to treat the adverse secondary effects arising from the comorbid disease to the SARS-CoV-2 human interactome^1^ or GWAS risk genes^2^ data. Here, we employ a raw score cutoff rather than the Z_d_ in eq. 1 for predicting an effective drug. A Z_d_ >1.65 cutoff will always give predictions regardless of the raw score. This potentially gives more false positives but could increase coverage for some low raw score predictions. Here, we use a raw score cutoff of 0.3 corresponding to ~30% precision from a benchmarking study that predicts if a drug is efficacious to a given disease. This empirical cutoff of 0.3 is based on determining a balance between the precision and the coverage (percentage of drugs having indication predictions). The coverage is ~60% with a 0.3 cutoff, whereas a larger cutoff, e.g., 0.4 will result in ~ 30% coverage while the precision only increases to ~40%. Drugs are ranked by the predicted precision weighted frequency of associated comorbid indications.

**CoVLS**

Here, we define a p-value weighted frequency of an input MOA as follows: If MOA protein T is shared by an indication D and the p-value of T associated with D is P, a weight of min(1.0,-$\alpha\log P$) is counted as T’s frequency (p-value<$e^{-1/\alpha}$will have a weight of 1 and p-value of 0.05 has a weight of around 3$\alpha$). In practice, we used cancer cell line data to optimize the coefficient $\alpha$(see below). An efficacious drug for a given COVID-19 secondary adverse event is predicted as follows: For all input proteins ranked by their comorbidity p-value weighted frequency, we screen them against FDA-approved drugs using target-based **FINDSITE^comb2.0^**. We then rank the collected drugs using the summation over all MOAs of a score defined as product of the predicted molecule’s binding precision times the p-value weighted frequency that the protein is a MOA of the comorbid secondary effect divided by the summed binding precision of all its targets. This ranking not only considers how likely a drug binds (precision) to a protein target and how likely the target is a MOA protein driver (by p-value weighted comorbidity frequency), but also the drug’s total number of targets or promiscuity (sum of all targets’ precision). If a drug is too promiscuous and has only a small number of MOA targets, its rank to a given target will be diminished. Thus, a high-ranking drug typically has multiple MOA targets with high p-value weighted comorbidity frequency with respect to the disease of interest.

**Validation of drug selection procedure**

To show that the above strategy of selecting efficacious drugs works in practice, we tested the two drug ranking procedures on ten cancer cell lines including breast cancer, ovarian cancer, colon cancer in the NCI-60 tumor human cell lines^33^. We applied **LeMeDISCO** to find the comorbid diseases of each cancer type and then took the top 100 comorbid diseases to rank the input MOAs of the disease of interest. We next screened the NCI diversity set of 1,597 molecules (https://dtp.cancer.gov/organization/dscb/obtaining/available_plates.htm) by **MEDICASCY**’s^4^ indication-based approach and by **FINDSITE^comb2.0^**’s^7^ target-based approach. Considering the top 20 ranked molecules using the two drug ranking approaches, success is defined as an IC50/IG50< 10^-4^ M response in the cell lines from the NCI-60 data^33^ for cell line growth inhibition. By varying the parameter $\alpha$ from 0.005 to 0.25, we set $\alpha$=0.025 which gives the best **CoVLS** mean success rate of 85.7%. A uniform weight of 1 for the comorbidity frequency will have a **CoVLS** mean success rate of 71.9%. Thus, the weighting method boosts **CoVLS** performance. The mean success rate from **CoMOAdrug** is 79.4% compared to 78.9% from **MEDICASCY**. While **CoMOAdrug** has close performance with **MEDICASCY**, it provides a way of targeting diseases not presented in **MEDICASCY’s** library**.** We note that CoVLS provides specific MOA targets, whereas **CoMOAdrug** does not. Another advantage of **CoVLS** over **CoMOAdrug** is that **FINDSITE^comb2.0^** is much faster than **MEDICASCY.** This is important when an extremely large compound library needs to be screened.

The above cell line tests are easier to analyze than those for human indications, since a predicted repurposed drug might be effective for a given indication; yet there are not any published results that show that this is the case. To benchmark test drugs directly for indications, we utilized the 2,059 training drugs with 123,146 drug-indications pairs in a modified jackknife test: For each drug, when predicting its indications, we use models trained from the other drugs having a Tanimoto Coefficient T_c_ < 0.8 to the given drug. This modified jackknife test avoids the naïve effects of similar drugs in the training set. We then evaluate the top 20 drug predictions for each indication using the above two strategies and compare the results to the single indication based **MEDICASCY** predictions^4^.

**MEDICASCY’s** drug efficacy prediction was benchmarked against the Project Rephetio method of Himmelstein et al.^20^ where a systematic integration of biomedical knowledge was used for computing drug features and a logistic regression machine learning was employed for learning and prediction. **MEDICASCY** has a significantly better AUPR for three benchmark sets: 0.172 for DrugCentral, 0.188 for ClinicalTrialSlim and 0.116 for the Symptomatic set, as compared to 0.056, 0.093, and 0.005, respectively by Project Rephetio of Himmelstein et al.^20^. Thus, comparison to **MEDICASCY** gives an indirect comparison of **CoMOAdrug** and **CoVLS** to the most similar alternative prediction method^20^.

For a given indication, experimental tests of the predicted top 20 drugs is well beyond the scope of this work. Thus, we only evaluate drugs having drug-indication relations in the **MEDICASCY** training set. In an earlier work^11^, we argued that for an incomplete list of drugs for a given indication of which a fraction $\gamma<1 \mathrm{of}$ drugs of the complete list are known to have that indication, the calculated precision $P_{cal}$ of drugs defined as (# of true positives within top 20)/20 for an indication is $P_{cal}=\gamma P$, where $P$ is the true prediction precision of the method and $\gamma\leq1$. In practice, since we do not know $\gamma$, we can only estimate the lower bound of $P=\frac{P_{cal}}{\gamma}\geq{maximal(P}_{cal}).$We assume that for a given indication, the larger the known number of drugs for that indication is (which of course are not used in the prediction component of the algorithm), the closer $\gamma$ is to 1.

In Table S5, we present the dependence of the calculated precision of each method on the cutoffs of the number of drugs with the given indication. Here, the optimized α=0.025 is used for CoVLS. Clearly, when the number of known drugs increases, $P_{cal}$ also increases. The maximal $P_{cal}$ of each method gives the closest lower bound for . Consistent with above cell line test, **CoVLS** has the best lower bound precision of 72.6%, **MEDICASCY**^34^ has the smallest lower bound precision of 58.0%, and **CoMOAdrug** is in-between, having a lower bound precision of 64.2%. All of these maximal values are at a cutoff of 170 drugs. After that cutoff, the calculated precisions have only slight fluctuations around the maximal values. On the other hand, for a known set of ~ 200 drugs, a random selection method can only have ~ 10% precision within the top 20. The mean enrichment factors within the top 20 by **CoMOAdrug** and **CoVLS** are 7.05 and 7.97, respectively. This benchmarking clearly demonstrates that the identification of efficacious drugs based on comorbidity indications for **CoMOAdrug** and prioritizing targets by their weighted comorbid frequency for **CoVLS** is a promising approach.

**Prediction of the top 20 drugs that might treat the severe adverse consequences of COVID-19**

In Tables S1 and S2, we presented the top 20 drugs for each of the complications having mapped comorbid indications from the SARS-CoV-2 human interactome^1^ or GWAS^2,3^ inputs, respectively. For human interactome input, 436 of 916 significant comorbidities (p-value <0.05) were mapped to 21 complications. For GWAS input, 313 of 598 significant comorbidities were mapped to 21 complications. For all complications, except for Dermatologic and Ocular, drugs of Dermatologic, Ophthalmological and insecticides were filtered out by their ATC classes^35^. Drugs with side effects predicted by **MEDICASCY** which is the same as the complications were excluded. For complications having multiple comorbid indications, drugs are ranked by the summed score over all indication’s ranking score.


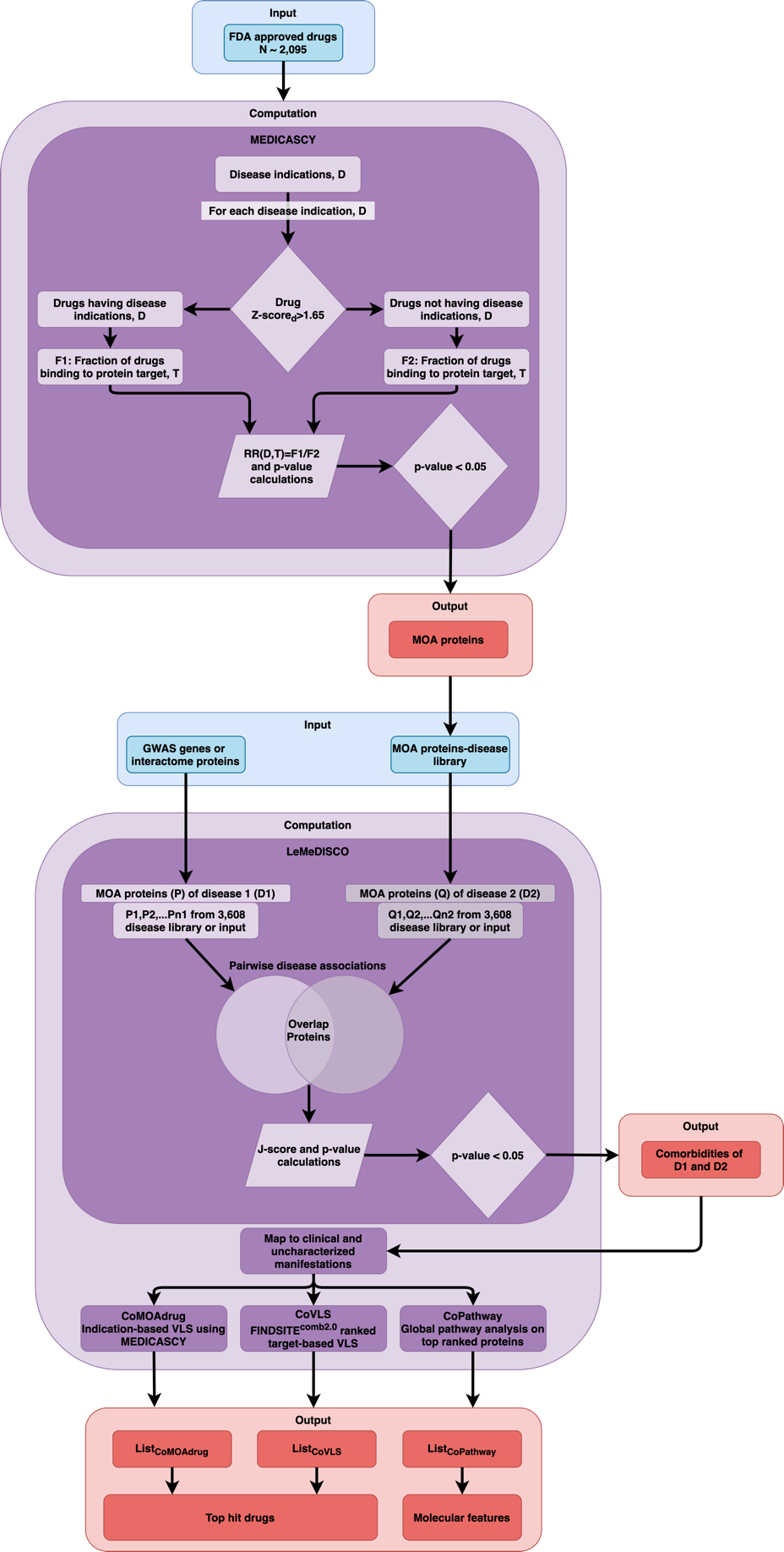


Fig. S1. Flowchart of MOATAI-VIR.


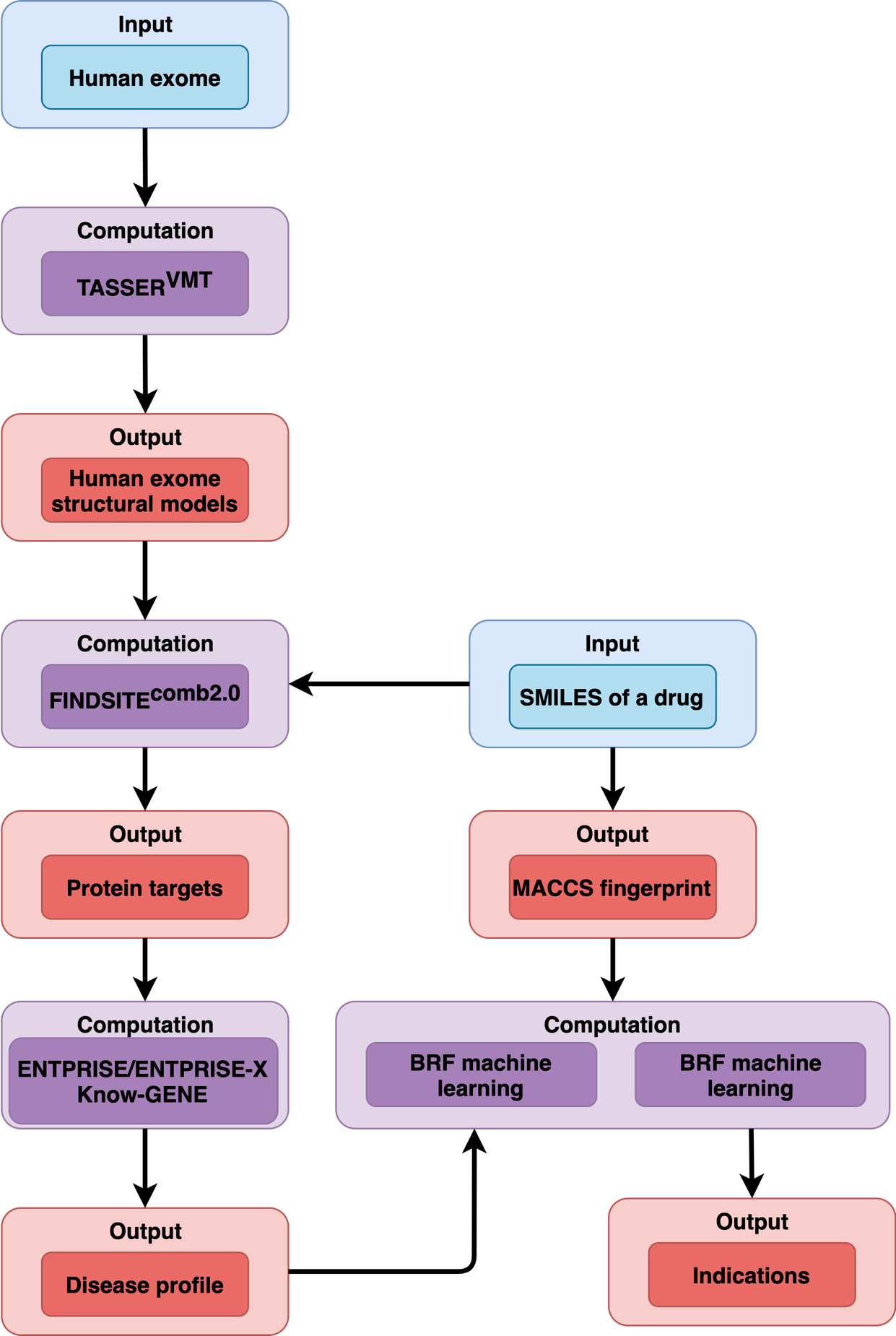


Fig. S2. Flowchart showing how indications are predicted by MEDICASCY.

Fig. S3. Flowchart showing how MEDICASCY predicts the MOA proteins associated with a given disease.


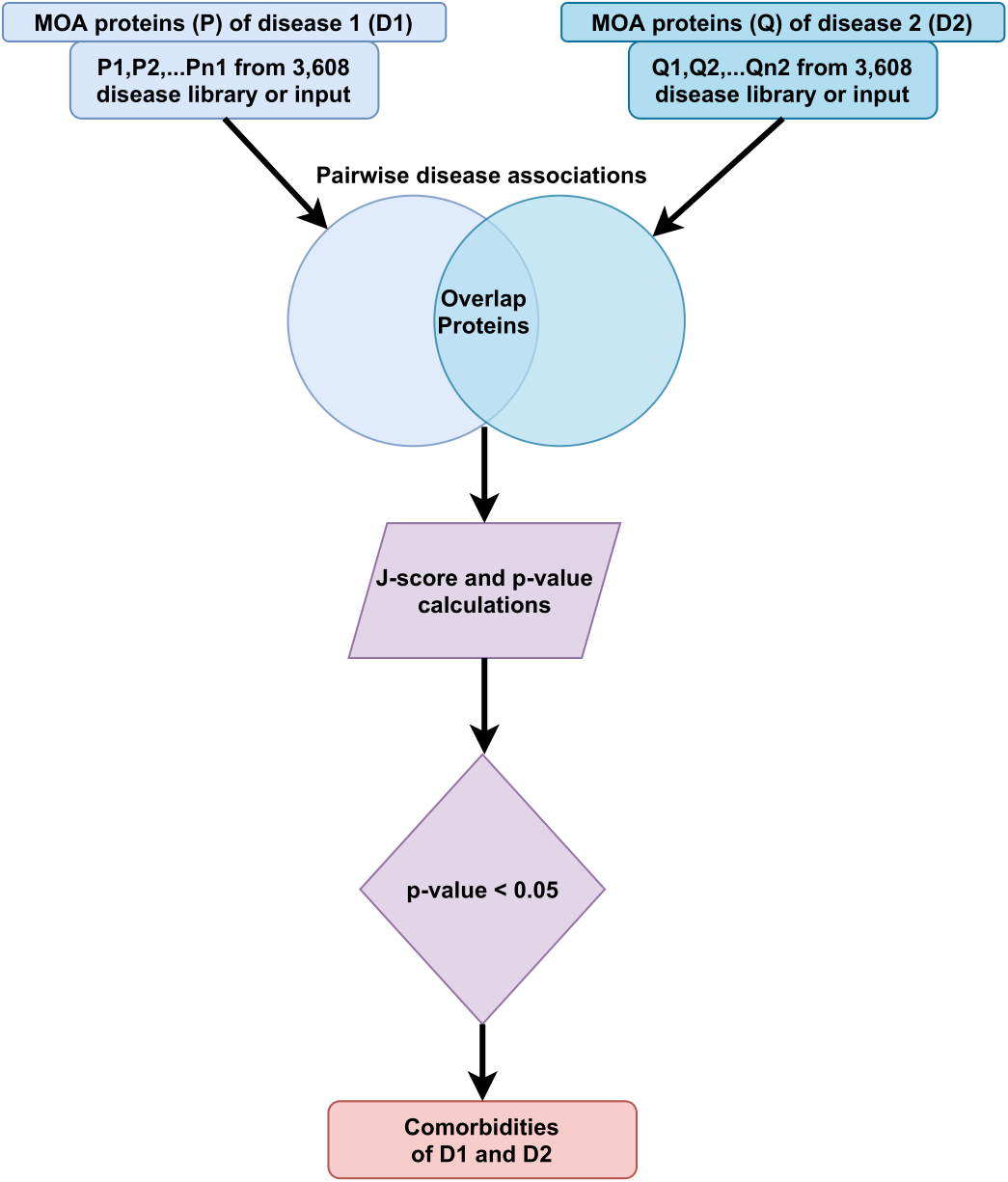


Fig. S4. LeMeDISCO J-score and p-value (see eq. 3) based protocol for determining the comorbidity of a given pair of diseases.

**Table S0**. Comparison of **LeMeDISCO**’s J-score with the XD score, NG, *S_AB_* score and symptom similarity for correlations with comorbidity quantified by the log(RR) score, φ**-**score and recall^a^.

|  | Unbinned^b^ | | 10 bins^b^ | | Recall |
| --- | --- | --- | --- | --- | --- |
|  | Log(RR) score | φ**-**score | Log(RR) score | φ**-**score |  |
| 198,149 pairs^c^ | | | | | |
| LeMeDISCO | **0.312(0.0)** | **0.218(0.0)** | **0.933(8.1 x 10^-5^)** | **0.900(3.9 x 10^-4^)** | **49.7%** |
|  | | | | | |
| 29,783 pairs^d^ | | | | | |
| LeMeDISCO | **0.185(0.0)** | **0.138(0.0)** | **0.939(5.6 x 10^-5^)** | **0.829(3.0 x 10^-3^)** | **56.0%** |
| XD score^26^ | 0.050(5.9 x 10^-18^) | 0.082(0.0) | 0.445(0.20) | 0.252(0.48) | 6.5% |
| NG^e^ | 0.008(0.17) | 0.058(1.3 x 10^-23^) | -0.436 | -0.175 | - |
|  | | | | | |
| 947 pairs^f^ | | | | | |
| LeMeDISCO | **0.217(1.5 x10^-11^)** | **0.282(9.0 x10^-19^)** | **0.682(0.030)** | **0.688(0.028)** | **75.8%** |
| *S_AB_* score^28^ | -0.188(5.5 x10^-9^) | -0.218(1.2 x10^-11^) | -0.671(0.034) | -0.473(0.17) | 8.5% |
|  | | | | | |
| 2,630 pairs^g^ | | | | | |
| LeMeDISCO | 0.184(1.9x10^-21^) | 0.196(3.5x10^-24^) | 0.774(8.6 x10^-3^) | 0.654(0.040) | 71.1% |
| Symptom similarity^27^ | **0.337(0.0)** | **0.197(1.6 x10^-24^)** | **0.950(2.6 x10^-5^)** | **0.960(1.1 x10^-5^)** | **100%** |

^a^ Numbers in parenthesis are the p-values of the corresponding correlation. Bold indicates the best results for the given data set.

^b^ Unbinned means raw data; each pair is a data point. 10 bins: partitioning the prediction scores into 10 equal size bins. In each bin, the Log(RR) & φ**-**score are averaged over data points in the bin. This gives equal weight to the rare prediction scores in the correlation analysis.

^c^ Mapping the DOID IDs from the human DO database to ICD9 IDs of Ref.^25^, gives a set of 198,149 disease pairs

^d^ Mapped the ICD9 disease code to our DOID of DO and obtained a consensus subset of 29,783 pairs from Table S0 dataset of 97,665 pairs in Ref.^26^.

^e^ NG is the number of shared genes between disease pairs in Ref.^26^.

^f^ Consensus set of 947 disease pairs from the dataset of Ref.^28^ and our dataset of 198,149.

^g^ A consensus dataset of 2,630 disease pairs was obtained from their Supplementary dataset 4 of Ref.^27^ compared to our set of 198,149 pairs.

**Tables S1-S4, S6-S8** are provided in the attached Excel spread sheets, but the Table titles are provided here for the convenience of the reader.

**Table S1.** Hierarchically ranked comorbidities, comorbidity enriched MOA proteins, pathways and the top 20 drugs for each COVID-19 clinical manifestation from the SARS-CoV-2 interactome^1^ as input results.

**Table S2.** Hierarchically ranked comorbidities, comorbidity enriched MOA proteins, pathways and the top 20 drugs for each COVID-19 clinical manifestation from the GWAS risk genes^2^ as input results.

**Table S3.** Hierarchically ranked comorbidities, comorbidity enriched MOA proteins, and the pathways for each uncharacterized COVID-19 manifestation from the SARS-CoV-2 interactome^1^ as input results.

**Table S4.** Hierarchically ranked comorbidities, comorbidity enriched MOA proteins, and pathways for each uncharacterized COVID-19 manifestation from the GWAS risk genes^2^ as input results.

**Table S6.** Interactome^1^ as input predicted neoplasm comorbidity enriched MOA proteins that

are labeled as cancer associated in the COSMIC^36^ database.

**Table S7.** GWAS risk genes^2^ as input predicted neoplasm comorbidity enriched MOA proteins that are labeled as cancer associated in the COSMIC^36^ database.

**Table S8.** COVID-19 differentially expressed genes from^37^ ranked by their adjusted p-value that are mapped to the COSMIC^36^ database.

**Table S5.** Dependence of calculated precision/enrichment factor of **CoMOAdrug** and **CoVLS** for the top 20 drugs on the cutoff of the number of known drugs associated with a disease

| Drug # cutoff | # of evaluated indications | MEDICASCY^a^ | CoMOAdrug | CoVLS |
| --- | --- | --- | --- | --- |
| 20 | 1029 | 0.273/3.10 | 0.270/4.53 | 0.305/5.03 |
| 30 | 891 | 0.309/3.48 | 0.304/4.61 | 0.346/5.29 |
| 40 | 790 | 0.335/3.62 | 0.332/4.52 | 0.376/5.13 |
| 50 | 710 | 0.364/3.94 | 0.360/4.60 | 0.408/5.27 |
| 60 | 675 | 0.377/4.10 | 0.374/4.67 | 0.425/5.37 |
| 70 | 652 | 0.386/4.20 | 0.383/4.71 | 0.434/5.38 |
| 80 | 602 | 0.406/4.44 | 0.407/4.89 | 0.464/5.66 |
| 90 | 458 | 0.487/5.43 | 0.516/5.96 | 0.575/6.59 |
| 100 | 423 | 0.509/5.63 | 0.546/6.18 | 0.612/6.90 |
| 110 | 404 | 0.521/5.78 | 0.563/6.31 | 0.634/7.10 |
| 120 | 384 | 0.536/5.93 | 0.586/6.51 | 0.659/7.32 |
| 130 | 370 | 0.549/6.05 | 0.601/6.65 | 0.678/7.49 |
| 140 | 364 | 0.555/6.12 | 0.609/6.72 | 0.685/7.56 |
| 150 | 356 | 0.561/6.17 | 0.617/6.79 | 0.696/7.66 |
| 160 | 347 | 0.568/6.23 | 0.628/6.90 | 0.708/7.78 |
| 170 | 336 | **0.580/6.35** | **0.642/7.05** | **0.726/7.97** |
| 180 | 228 | 0.572/6.05 | 0.623/6.61 | 0.715/7.61 |
| 190 | 106 | 0.558/5.57 | 0.591/5.91 | 0.673/6.78 |
| 200 | 51 | 0.537/5.04 | 0.573/5.38 | 0.620/5.90 |

**^a^ MEDICASCY** results are included for comparison. Bold number is the maximal calculated precision (1^st^ number) of each method. For a given testing drug, any drugs with Tanimoto Coefficient > 0.8 to it are excluded from training. Enrichment factor (2^nd^ number) is defined as the following ratio: (number of predicted true positives within the top 20)/(number of true positives by randomly selecting 20 drugs from the 2,059 drugs).

**Table S9.** Statistical significance (p-value) of virus human interacting proteins’ overlap with the 723 COSMIC cancer drivers^36^

| Virus | Reference of interactome | # of interactome proteins | # of Neoplasm comorbid indications within top 100 | p-value of all interactome protein overlap | p-value of overlap of top 100 proteins after LeMeDISCO prioritization |
| --- | --- | --- | --- | --- | --- |
| SARS-CoV-2 | Ref.^1^ | 332 | 56 | 0.10 | 0.016 |
|  | | | | | |
| Known oncoviruses | | | | | |
| Ad5 | Ref.^38^ | 153 | 10 | 0.08 | 0.35 |
| EBV | Ref.^38^ | 752 | 27 | 0.0040 | 1.0$\times{10}^{-13}$ |
| PyV | Ref.^38^ | 314 | 21 | 3.0$\times{10}^{-4}$ | 0.04 |
| HBV | Ref.^39^ | 146 | 51 | $1.9\times{10}^{-24}$ | $1.3\times{10}^{-20}$ |
| HCV | Ref.^40^ | 617 | 72 | $9.2\times{10}^{-10}$ | $1.3\times{10}^{-22}$ |
| HIV | Ref.^41^ | 4092 | 65 | $2.7\times{10}^{-24}$ | 0.04 |
| HPV | Ref.^42^ | 2049 | 89 | $2.3\times{10}^{-18}$ | $1.3\times{10}^{-20}$ |
| KSHV | Ref.^43^ | 556 | 46 | $8.3\times{10}^{-4}$ | 0.19 |
| HTLV | Ref.^44^ | 122 | 6 | 0.10 | 0.55 |
|  | | | | | |
| Known non oncoviruses | | | | | |
| Zaire-EBOLA | Ref.^45^ | 250 | 17 | $1.5\times{10}^{-4}$ | 0.19 |
| H1N1 | Ref.^46^ | 87 | 3 | 0.66 | 0.75 |
| DENV | Ref.^47^ | 222 | 20 | $8.4\times{10}^{-6}$ | 0.19 |
| HSV1 | Ref.^48^ | 606 | 34 | $5.0\times{10}^{-5}$ | 0.19 |

**SI References**

1 Gordon, D. E. *et al.* A SARS-CoV-2 protein interaction map reveals targets for drug repurposing. *Nature*, doi:10.1038/s41586-41020-42286-41589, doi:10.1038/s41586-020-2286-9 (2020).

2 Ellinghaus, D., et al. & The_Severe_Covid-19_GWAS_Group. Genomewide Association Study of Severe Covid-19 with Respiratory Failure. *N. Engl. J. Med.*, doi: 10.1056/NEJMoa2020283 (2020).

3 Wu, C. *et al.* Risk Factors Associated With Acute Respiratory Distress Syndrome and Death in Patients With Coronavirus Disease 2019 Pneumonia in Wuhan, China. *JAMA Intern Med*, doi:10.1001/jamainternmed.2020.0994, doi:10.1001/jamainternmed.2020.0994 (2020).

4 Zhou, H. *et al.* MEDICASCY: A Machine Learning Approach for Predicting Small Molecule Drug Side Effects, Indications, Efficacy and Mode of Action. *Mol Pharm* **17**, 1558-1574, doi:10.1021/acs.molpharmaceut.9b01248 (2020).

5 Jaccard, P. THE DISTRIBUTION OF THE FLORA IN THE ALPINE ZONE. *New Phytologist* **11**, 37-50 (1912).

6 Fisher, R. A. On the Interpretation of χ 2 from Contingency Tables, and the Calculation of P. *Journal of the Royal Statistical Society* **85**, 87-94 (1922).

7 Zhou, H., Cao, H. & Skolnick, J. FINDSITE^comb2.0^: A New Approach for Virtual Ligand Screening of Proteins and Virtual Target Screening of Biomolecules. *Journal of Chemical Information and Modeling* **58**, 2343-2354 (2018).

8 Wishart, D. *et al.* DrugBank: a comprehensive resource for in silico drug discovery and exploration. . *Nucl. Acid. Res.* **34**, D668-672 (2006).

9 Zhou, H. & Skolnick, J. Template-based protein structure modeling using TASSER^VMT^. *Proteins* **80**, 352-361 (2012).

10 Zhou, H. & Skolnick, J. FINDSITE^X^: A Structure-Based, Small Molecule Virtual Screening Approach with Application to All Identified Human GPCRs. *Molecular Pharmaceutics* **9**, 1775-1784 (2012).

11 Zhou, H., Gao, M. & Skolnick, J. Comprehensive prediction of drug-protein interactions and side effects for the human proteome. *Scientific Reports* **5**, 11090 (2015).

12 Wild, D. J. & Blankley, C. J. Comparison of 2D fingerprint types and hierarchy level selection methods for structural grouping using Ward's clustering. *J Chem Inf Comput Sci* **40**, 155-162 (2000).

13 Zhou, H., Gao, M. & Skolnick, J. ENTPRISE: An Algorithm for Predicting Human Disease-Associated Amino Acid Substitutions from Sequence Entropy and Predicted Protein Structures. *PLoS One* **11**, e0150965, doi:10.1371/journal.pone.0150965 (2016).

14 Zhou, H., Gao, M. & Skolnick, J. ENTPRISE-X: Predicting disease-associated frameshift and nonsense mutations. *PLoS One* **13**, e0196849, doi:10.1371/journal.pone.0196849 (2018).

15 Zhou, H. & Skolnick, J. A knowledge-based approach for predicting gene-disease associations. *Bioinformatics* **32**, 2831-2838, doi:10.1093/bioinformatics/btw358 (2016).

16 Ho, T. K. in *Proceedings of the 3rd International Conference on Document Analysis and Recognition.* 278-282.

17 Lynn Marie Schriml, C. A., Suvarna Nadendla, Yu-Wei Wayne Chang, Mark Mazaitis, Victor Felix, Gang Feng, Warren Alden Kibbe. Disease Ontology: a backbone for disease semantic integration. *Nucleic Acids Research* (2012).

18 Y. H. Li *et al.* Therapeutic target database update 2018: enriched resource for facilitating bench-to-clinic research of targeted therapeutics. *Nucleic Acids Res.* **46**, D1121-D1127 (2018).

19 Kuhn, M., Campillos, M., Letunic, I., Jensen, L. & Bork, P. A side effect resource to capture phenotypic effects of drugs. *Mol Syst Biol.* **6**, 343 (2010).

20 Himmelstein, D. S. *et al.* Systematic integration of biomedical knowledge prioritizes drugs for repurposing. *eLife* **6**, e26726 (2017).

21 Tanimoto, T. T. An Elementary Mathematical Theory of Classification and Prediction. *IBM Interanl Report* (1958).

22 Mehta, C. R. & Patel, N. R. ALGORITHM 643: FEXACT: a FORTRAN subroutine for Fisher's exact test on unordered r×c contingency tables. *ACM Transactions on Mathematical Software* **12**, 154-161 (1986).

23 Uhlén, M. *et al.* Tissue-based map of the human proteome. *Science* **347**, 1260419, doi:10.1126/science.1260419 (2015).

24 Hintzsche, J. D., Robinson, W. A. & Tan, A. C. A Survey of Computational Tools to Analyze and Interpret Whole Exome Sequencing Data. *International Journal of Genomics* **2016**, 7983236, doi:10.1155/2016/7983236 (2016).

25 Hidalgo, C. A., Blumm, N., Barabasi, A. L. & Christakis, N. A. A dynamic network approach for the study of human phenotypes. *PLoS computational biology* **5**, e1000353 (2009).

26 Ko, Y., Cho, M., Lee, J.-S. & Kim, J. Identification of disease comorbidity through hidden molecular mechanisms. *Scientific Reports* **6**, 39433 (2016).

27 Zhou, X., Menche, J., Barabási, A.-L. & Sharma, A. Human symptoms–disease network. *Nature Communications* **5**, 4212 (2014).

28 Menche, J. *et al.* Disease networks. Uncovering disease-disease relationships through the incomplete interactome. *Science* **347**, 1257601 (2015).

29 Rogers, F. B. Medical subject headings. *Bull Med Libr Assoc* **51**, 114-116 (1963).

30 Schriml, L. M. *et al.* Human Disease Ontology 2018 update: classification, content and workflow expansion. *Nucleic Acids Res* **47**, D955-D962, doi:10.1093/nar/gky1032 (2019).

31 World Health Organization. (World Health Organization, 2004).

32 Jassal, B. *et al.* The reactome pathway knowledgebase. *Nucleic Acids Res* **48**, D498-d503, doi:10.1093/nar/gkz1031 (2020).

33 NCI-60 Human Tumor Cell Lines Screen. [*https://dtp.cancer.gov/discovery_development/nci-60/*](https://dtp.cancer.gov/discovery_development/nci-60/)

34 Zhou, Y., Wang, F., Tang, J., Nussinov, R. & Cheng, F. Artificial intelligence in COVID-19 drug repurposing. *The Lancet Digital Health* **2**, e667-e676 (2020).

35 Skrbo A, B. B., Skrbo S. Classification of drugs using the ATC system (Anatomic, Therapeutic, Chemical Classification) and the latest changes. *Medicinski Arhiv* **58** (2004).

36 Tate, J. G. *et al.* COSMIC: the Catalogue Of Somatic Mutations In Cancer. *Nucleic Acids Research* **47**, D941-D947, doi:10.1093/nar/gky1015 (2019).

37 Blanco-Melo, D. *et al.* Imbalanced Host Response to SARS-CoV-2 Drives Development of COVID-19. *Cell* **181**, 1036-1045.e1039, doi:10.1016/j.cell.2020.04.026 (2020).

38 Rozenblatt-Rosen, O. *et al.* Interpreting cancer genomes using systematic host network perturbations by tumour virus proteins. *Nature* **487**, 491-495, doi:10.1038/nature11288 (2012).

39 Wu, Z. J., Zhu, Y., Huang, D. R. & Wang, Z. Q. Constructing the HBV-human protein interaction network to understand the relationship between HBV and hepatocellular carcinoma. *J Exp Clin Cancer Res* **29**, 146, doi:10.1186/1756-9966-29-146 (2010).

40 Kwofie, S. K., Schaefer, U., Sundararajan, V. S., Bajic, V. B. & Christoffels, A. HCVpro: Hepatitis C virus protein interaction database. *Infection, Genetics and Evolution* **11**, 1971-1977, doi:<https://doi.org/10.1016/j.meegid.2011.09.001> (2011).

41 NCBI. <<https://www.ncbi.nlm.nih.gov/genome/viruses/retroviruses/hiv-1/interactions/browse/>> (

42 Farooq, Q. u. A. *et al.* Inferring Virus-Host relationship between HPV and its host Homo sapiens using protein interaction network. *Scientific Reports* **10**, 8719, doi:10.1038/s41598-020-65837-w (2020).

43 Davis, Z. H. *et al.* Global mapping of herpesvirus-host protein complexes reveals a transcription strategy for late genes. *Mol Cell* **57**, 349-360, doi:10.1016/j.molcel.2014.11.026 (2015).

44 Simonis, N. *et al.* Host-pathogen interactome mapping for HTLV-1 and -2 retroviruses. *Retrovirology* **9**, 26, doi:10.1186/1742-4690-9-26 (2012).

45 Muthaiyan, M., Pushan, S. S., Naorem, L. D. & Venkatesan, A. Understanding of Zaire ebolavirus-human protein interaction for drug repurposing. *Virusdisease* **31**, 28-37, doi:10.1007/s13337-020-00570-6 (2020).

46 Shapira, S. D. *et al.* A physical and regulatory map of host-influenza interactions reveals pathways in H1N1 infection. *Cell* **139**, 1255-1267, doi:10.1016/j.cell.2009.12.018 (2009).

47 Amemiya, T., Gromiha, M. M., Horimoto, K. & Fukui, K. Drug repositioning for dengue haemorrhagic fever by integrating multiple omics analyses. *Scientific Reports* **9**, 523, doi:10.1038/s41598-018-36636-1 (2019).

48 Lian, X. *et al.* Prediction and analysis of human-herpes simplex virus type 1 protein-protein interactions by integrating multiple methods. *Quantitative Biology* **8**, 312-324, doi:10.1007/s40484-020-0222-5 (2020).
